# An immunomodulatory signature of responsiveness to immune checkpoint blockade therapy

**DOI:** 10.1002/ctm2.238

**Published:** 2020-12-21

**Authors:** Hongru Shen, Xilin Shen, Dan Wu, Mengyao Feng, Yichen Yang, Yang Li, Meng Yang, Wei Wang, Qiang Zhang, Fangfang Song, Kexin Chen, Xiangchun Li

**Affiliations:** ^1^ Tianjin Cancer Institute National Clinical Research Center for Cancer Key Laboratory of Cancer Prevention and Therapy Tianjin Medical University Cancer Institute and Hospital, Tianjin Medical University Tianjin China; ^2^ Department of Epidemiology and Biostatistics National Clinical Research Center for Cancer Key Laboratory of Cancer Prevention and Therapy Key Laboratory of Molecular Cancer Epidemiology of Tianjin Tianjin Medical University Cancer Institute and Hospital, Tianjin Medical University Tianjin China; ^3^ Department of Maxillofacial and Otorhinolaryngology Oncology National Clinical Research Center for Cancer Key Laboratory of Cancer Prevention and Therapy Tianjin Medical University Cancer Institute and Hospital Tianjin Medical University Tianjin China

Dear Editor,

We identified an immunomodulatory signature that was significantly associated with clinical improvement of immune checkpoint blockade (ICB) therapy response and better prognosis in urothelial carcinoma^1^ and melanoma.^2^ Our finding is helpful for identifying cancer patients who may benefit from ICB therapy.

ICB therapy is an effective treatment regimen for cancer patients. Tumor mutation burden (TMB),^3^ expression of *PD‐1*, *PD‐L1*,^4^ and *CTLA‐4*, are known factors associated with ICB therapy response. However, our understanding of biomarkers for response to ICB therapy remains incomplete. This necessitates further investigation on identification of reliable biomarkers for ICB therapy response.

A flowchart depicting procedures of the study was shown in Figure 1A, which consisted of feature representation learning of gene expression from single cells and association of expression signatures with ICB therapy response in two clinical trials. We collected gene expression data from 71,494 single cells encompassing tumor cells, stroma cells, dendritic cells, CD4+, and CD8+ T cells from seven cancer types (Table S1). A deep learning model^5^, ^6^ was developed by iteratively training on gene expression data of these 71,494 single cells. Subsequently, it was applied to extract 258 expression signatures for single cells and samples from two clinical trials. We ranked the extracted expression signatures of single cells with a gene set involved in immunomodulation^7^ (see Supporting Information Methods, Table S2) and examined association of the top five ranking signatures with clinical improvement of ICB therapy and prognosis.

**FIGURE 1:**
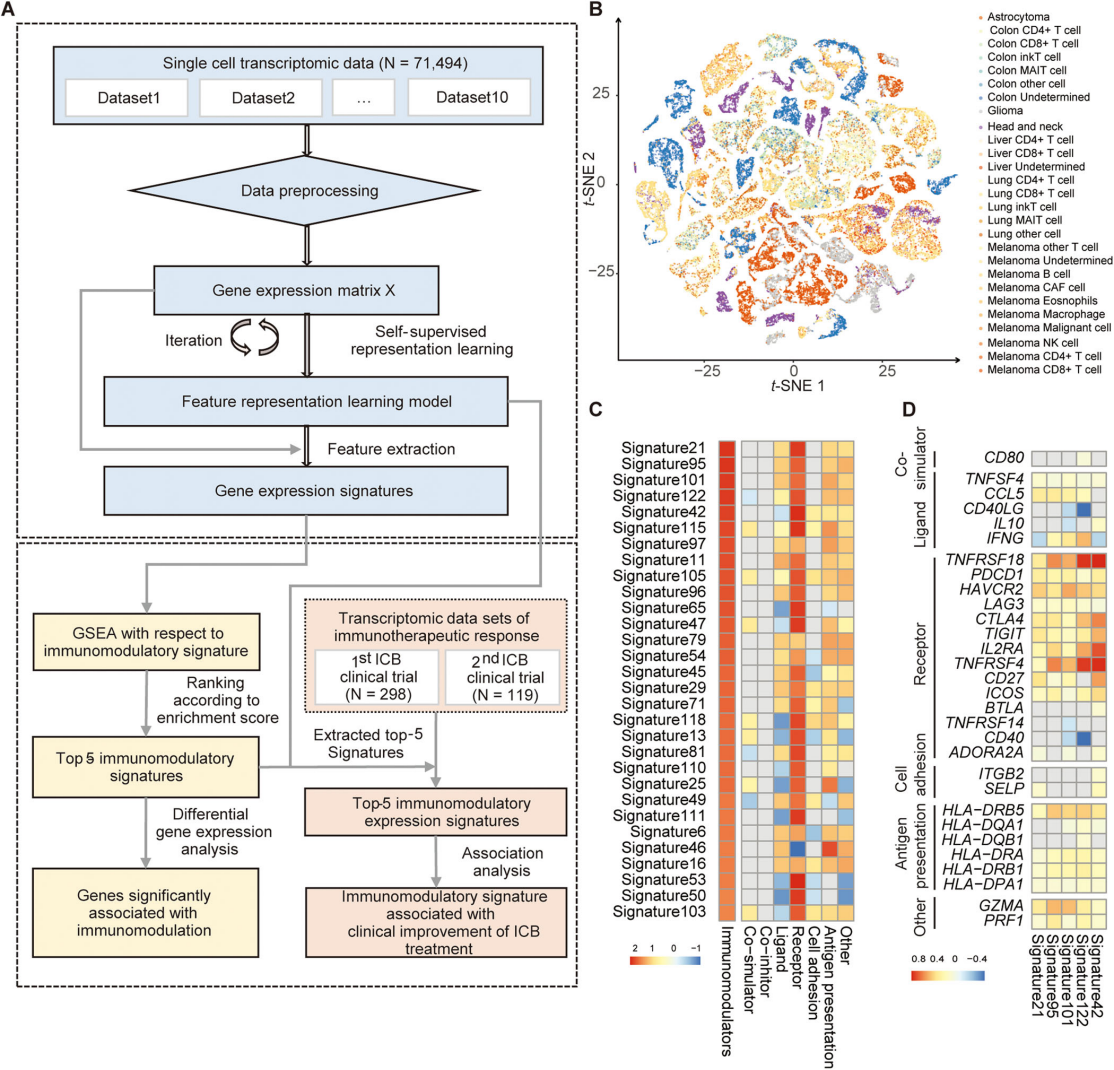
Flowchart depicting study procedures and gene expression signatures derived from deep learning model. This flowchart (A) consists of two components: (upper panel) development of deep learning model for extracting gene expression signatures (lower panel) identification of signatures associated with clinical improvement of ICB therapy. (B) Scatter plot depicting the grouping of single cells unveiled by deep learning. (C) Heatmap representation of the top‐ranking signatures with respect to immunomodulators and its subcategories: co‐simulator, co‐inhibitor, ligand, receptor, cell adhesion, antigen presentation, and others. (D) Association of genes with respect to immunomodulators stratified by its subcategories for the top five ranking signatures

The *t*‐SNE embedded scatter plot shown in Figure 1B depicted single cell clusters unveiled from expression signatures learned by deep learning model. The same cell types (such as CD4+ or CD8+ T cell) from different data set were clustered together, whereas different cell types were separated distinctively (Figures S1A and B). Notably, a cluster of unsorted melanoma single cells were interspersed with different T‐cell types (Figure S1C).

We observed that 63.6% (164/258) of expression signatures extracted from single cells were significantly associated with immunomodulation^7^ (Figure 1C), out of which the receptor subcategory was the most significantly enriched among seven groups. The top five ranking immunomodulatory signatures were characterized by upregulation of genes related to immunomodulatory ligands (e.g., *TNFSF4*, *CCL5*, and *IFNG*), receptors (e.g., *HAVCR2*, *TNFRSF18*, *PDCD1*, and *LAG3*), and antigen presentation (e.g., *HLA‐DRB5*, *HLA‐DRA*, and *HLA‐DRB1*) (Figure 1D).

We categorized ICB therapy response into clinical and no clinical improvement (see Supporting Information Methods). The baseline characteristics are shown in Table S3. The urothelial carcinoma clinical trial^1^ consisted of 298 urothelial cancer patients all treated with PD‐L1 inhibitor, 68 of which were categorized as clinical improvement and the rest 230 were categorized as no clinical improvement group. The melanoma clinical trial^2^ consisted of 119 melanoma cancer patients, 47 of which achieved clinical improvement and the rest did not. In melanoma clinical trial, 72 patients received PD‐1 inhibitor, whereas 47 patients received CTLA4 in conjunction with PD‐1 inhibitor. The other available molecular markers related to ICB therapy response or prognosis such as TMB, the degree of cytotoxic lymphocytes infiltration (CTL),^8^ expression levels of *PD‐1*, *PD‐L1*, and *CTLA4*, and clinical features are summarized in Table S2. TMB data were available for 78.5% (234/298) and 100% (119/119) of patients in these two clinical trials.

Among these top five ranking signature, one of the expression signature scores was significantly different between clinical improvement group and no clinical improvement group for the urothelial carcinoma (Figure 2A; −0.72 versus −0.09, 95% CI = −0.82 to −0.62 versus −0.30 to 0.12; Wilcoxon test, adjusted *P* < .001) and melanoma (Figure 2B; −0.69 versus −0.24, 95% CI = −0.88 to −0.50 versus −0.50 to 0.02; Wilcoxon test, adjusted *P* = .03) clinical trials. This immunomodulatory signature was significantly associated with clinical improvement of ICB therapy across two clinical trials (OR = 4.34 and 3.32, 95% CI = 2.30‐8.23 and 1.35‐8.47; Fisher's exact test, *P* < 0.001 and *P* = 0.006). Association of this immunomodulatory signature with clinical improvement of ICB therapy remained statistically significant after controlling for TMB, CTL, expression levels of *PD‐1*, *PD‐L1*, and *CTLA4* across the urothelial carcinoma clinical trial (OR = 1.30, 95% CI = 1.14‐1.47; multivariate logistic model, *P* < 0.001; Figure 2C) and melanoma clinical trial (OR = 1.32, 95% CI = 1.09‐1.59; multivariate logistic model, *P* = 0.005; Figure 2D). We further examined the significance of identified immunomodulatory signature with respect to anti‐PD1 and anti‐PD‐L1 inhibitors. In urothelial carcinoma clinical trial, all patients were treated with anti‐PD‐L1 inhibitors. In melanoma clinical trial, the identified immunomodulatory signature remained significant in patients treated with anti‐PD‐1 treatment (OR = 1.32, 95% CI = 1.05‐1.67, *P* = 0.02; Figure S2A), while it also trended toward better association in patients treated with anti‐PD‐1 in conjunction with anti‐CTLA4 (Figure S2B).

**FIGURE 2:**
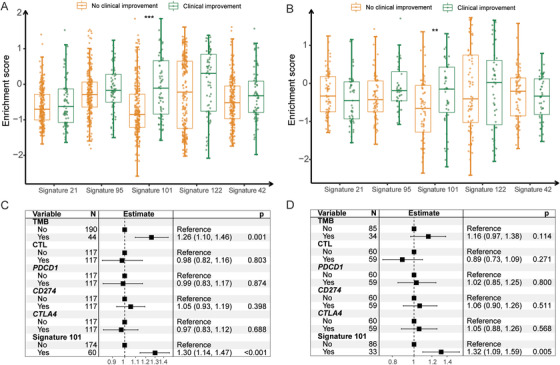
Association between immunomodulatory signatures and clinical improvement of ICB therapy. (A and B) Box plot representation of the differences between clinical improvement group and no clinical improvement of ICB therapy in top five immunomodulatory signatures in urothelial carcinoma and melanoma. The *P*‐values were subjected for multiple hypothesis test. (*** represented adjusted *P* ≤ .001 and ** .05 < adjusted *P* < .001). (C and D) Forest plot representation of association between the identified immunomodulatory signature and clinical improvement of cancer ICB therapy with other confounding factors taken into account in urothelial carcinoma and melanoma. The confounding factors included TMB, CTL, and expression levels of *PD‐1*, *PD‐L1*, and *CTLA‐4*. Adjusted *P*‐value < .05 was considered to be significant

In addition, the identified immunomodulatory signature was significantly associated with improved overall survival in urothelial carcinoma clinical trial (log‐rank test, *P* = 0.004; Figure 3A) and marginally significant in melanoma trial (log‐rank test, *P* = 0.07; Figure 3B). This identified signature remained significant in urothelial cohort (HR = 0.61, 95% CI = 0.39‐0.96; multivariate Cox model, *P* = 0.03; Figure 3C) and marginal significant in melanoma cohort (HR = 0.57, 95% CI = 0.30‐1.07; multivariate Cox model, *P* = 0.08; Figure 3D) after controlling for confounding factors.

**FIGURE 3:**
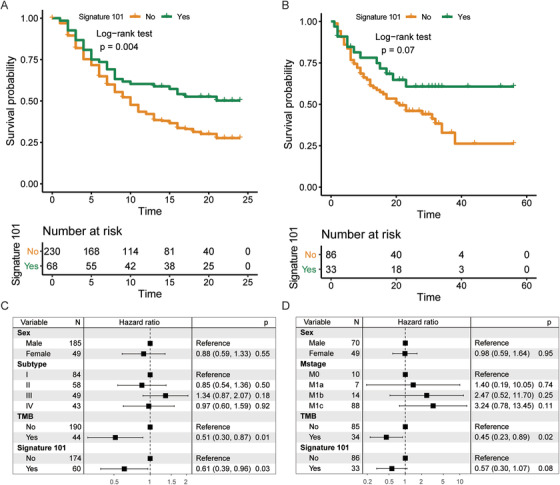
Association between the identified immunomodulatory signature and prognosis in two ICB therapy clinical trials. Kaplan‐Meier survival curves (A and B) and forest plot representation of multivariate Cox regression model (C and D) depicting the association between the identified immunomodulatory signature and prognosis in two ICB therapy clinical trials. The confounding factors include sex, TCGA molecular subtype, or melanoma stage and TMB. The age information was not available. Adjusted *P*‐value < .05 was considered to be significant while .05 ≤ adjusted *P*‐value ≤ .1 was considered to be marginally significant

Gene set enrichment analysis revealed that immune‐related signaling circuits^9^ (Table S4) were overrepresented in patients with activated immunomodulatory signature, while signatures featuring cancer cell proliferation and invasiveness such as epithelial‐to‐mesenchymal transition and angiogenesis were underrepresented (Figure S3). In addition, tumor immune microenvironment signatures obtained from CIBERSORT algorithm^10^ showed that there were significant differences among infiltration of CD8+ T cells, activated CD4+ memory T cells, M1 macrophages, and dendritic cells in patients stratified by this signature (Figure S4).

In summary, we reported that an immunomodulatory signature dissected from large‐scale single cell expression data was significantly associated with clinical improvement of ICB therapy and better prognosis in the two independent ICB therapy clinical trials. However, further investigation in prospective randomized clinical trial is warranted.

## ETHICS APPROVAL AND CONSENT TO PARTICIPATE

This study was approved by the institutional review board (IRB) of Tianjin Cancer Hospital. Informed consent was exempted by the IRB given that data were obtained from public, open access database.

## CONFLICT OF INTEREST

The authors declare that they have no conflict of interest.

## FUNDING INFORMATION

National Natural Science Foundation of China; Grant No. 31801117; Program for Changjiang Scholars and Innovative Research Team in University in China; Grant No.: IRT_14R40; Tianjin Science and Technology Committee Foundation; Grant No.: 17JCYBJC25300; Chinese National Key Research and Development Project; Grant No. 2018YFC1315600.

## AUTHOR CONTRIBUTIONS

Xiangchun Li and Kexin Chen designed and supervised the study; Hongru Shen and Xiangchun Li performed data collection, analysis, and wrote the manuscript; Dan Wu, Xilin Shen, Mengyao Feng, Yichen Yang, Yang Li, and Meng Yang collected data; Wei Wang and Qiang Zhang interpretated the data; Xiangchun Li, Kexin Chen, and Hongru Shen revised the manuscript.

## Supporting information

Figure S1. *t*‐SNE visualization of (A) CD4+ and CD8+ T cells, (B) astrocytoma, melanoma, and head and neck carcinoma, and (C) CD4+, CD8+ T cells, and unsorted admixed melanoma cells.
Figure S2. Association between the identified immunomodulatory signature and clinical improvement in (A) anti‐PD‐1 and (B) anti‐PD‐1 in conjunction with anti‐CTL4 from the melanoma ICB therapy clinical trial. Confounding factors included TMB, CTL and expression levels of *PD‐1*, *PD‐L1* and *CTLA‐4*.
Figure S3. Gene set enrichment analysis of the identified immunomodulatory signature. Signaling pathways statistically significant (adjusted p < 0.10) in both two datasets were displayed in the heatmap.
Figure S4. Tumor immune microenvironment signatures unveiled by CIBERTSORT. The infiltration difference of each immune signature was evaluated by t‐test. The p‐values were subjected for multiple hypothesis test. Adjusted p‐value < 0.05 was considered to be significant. (*** represented adjusted p ≤ 0.001 and ** 0.05 < adjusted p < 0.001)

**Table S1**. Data source information.
**Table S2**. Immunomodulatory gene sets.
**Table S3**. Clinical characteristic of the patients with ICB therapy in two clinical trials.
**Table S4**. Immune related signaling gene sets.

## Data Availability

Data are available in open access database. All data relevant to the study are included in the article or uploaded as supplementary information. All data generated or analyzed during this study are included in this manuscript. A website running the developed deep learning model to extract gene expression signatures is freely available at https://lixiangchun.github.io.
