# Supplementary material for: An immunomodulatory signature of responsiveness to immune checkpoint blockade therapy
Source: Clin Transl Med. 2020 Dec 21;10(8):e238. doi: 10.1002/ctm2.238 (PMC7752154; doi:10.1002/ctm2.238)
Supplement: Supplementary file 1 — Figure S1. t‐SNE visualization of (A) CD4+ and CD8+ T cells, (B) astrocytoma, melanoma, and head and neck carcinoma, and (C) CD4+, CD8+ T cells, and unsorted admixed melanoma cells. Figure S2. Association between the identified immunomodulatory signature and clinical improvement in (A) anti‐PD‐1 and (B) anti‐PD‐1 in conjunction with anti‐CTL4 from the melanoma ICB therapy clinical trial. Confounding factors included TMB, CTL and expression levels of PD‐1, PD‐L1 and CTLA‐4. Figure S3. Gene set enrichment analysis of the identified immunomodulatory signature. Signaling pathways statistically significant (adjusted p < 0.10) in both two datasets were displayed in the heatmap. Figure S4. Tumor immune microenvironment signatures unveiled by CIBERTSORT. The infiltration difference of each immune signature was evaluated by t‐test. The p‐values were subjected for multiple hypothesis test. Adjusted p‐value < 0.05 was considered to be significant. (*** represented adjusted p ≤ 0.001 and ** 0.05 < adjusted p < 0.001) [file CTM2-10-e238-s001.docx]

**SUPPLEMENTARY MATERIALS**

**
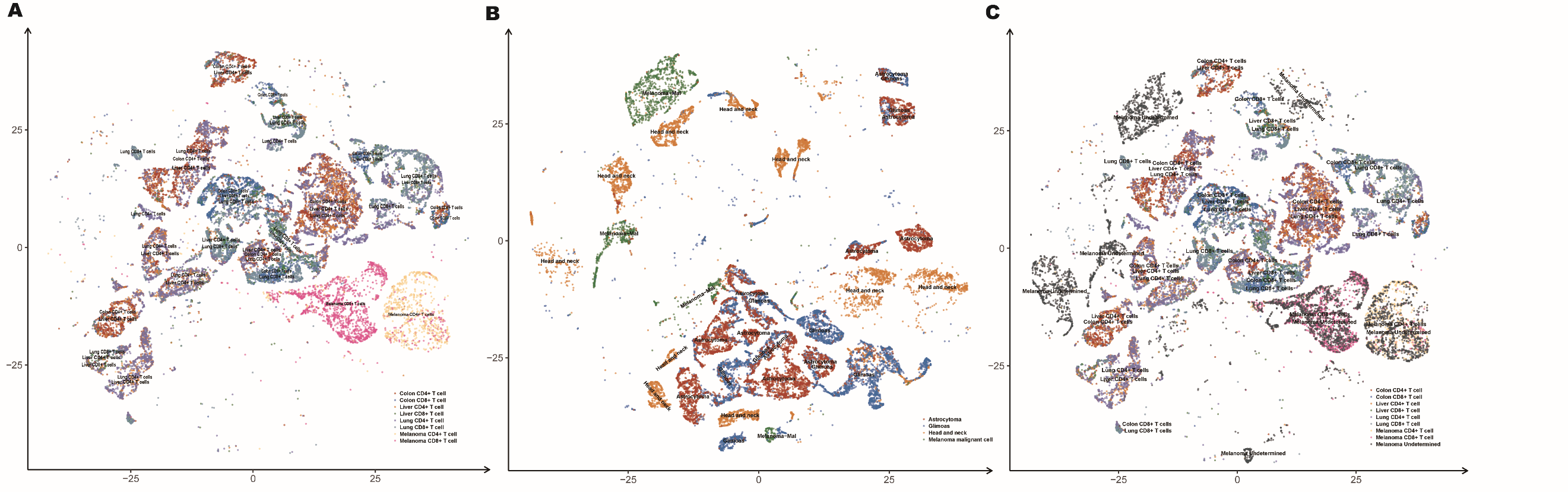
**

**Figure S1. *t*-SNE visualization of (A) CD4+ and CD8+ T cells, (B) Astrocytoma, melanoma and head and neck carcinoma, and (C) CD4+, CD8+ T cells and unsorted admixed melanoma cells.**


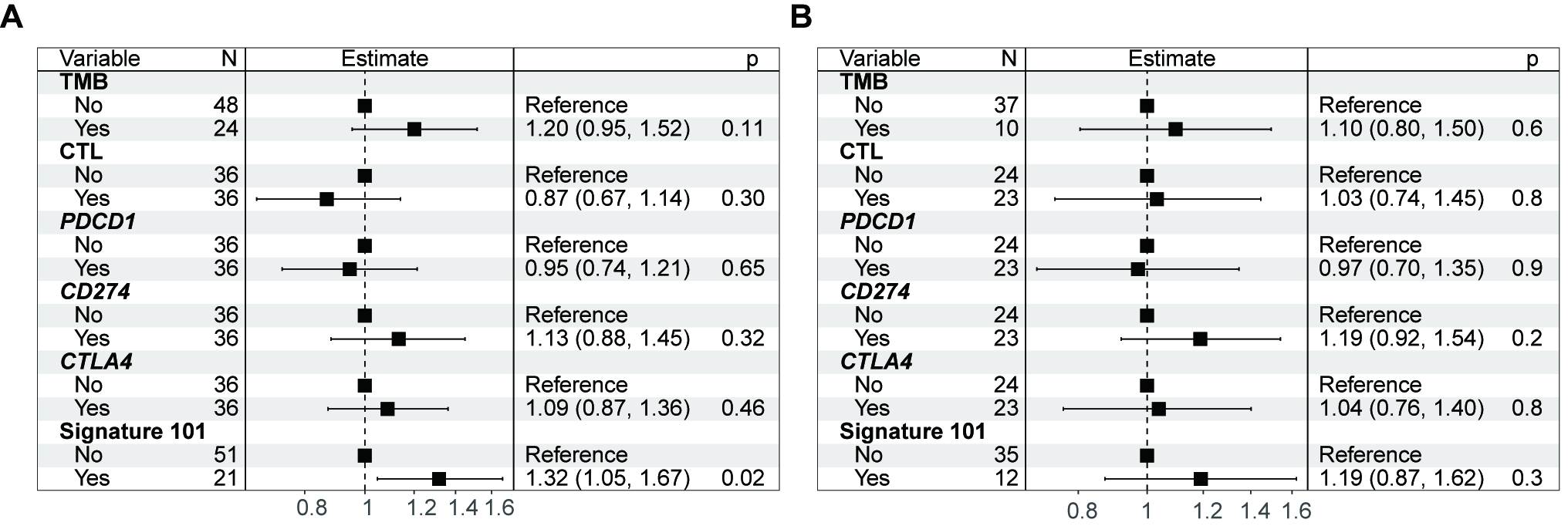


**Figure S2. Association between the identified immunomodulatory signature and clinical improvement in (A) anti-PD-1 and (B) anti-PD-1 in conjunction with anti-CTL4 from the melanoma ICB therapy clinical trial.** Confounding factors included TMB, CTL and expression levels of *PD-1*, *PD-L1* and *CTLA-4*.


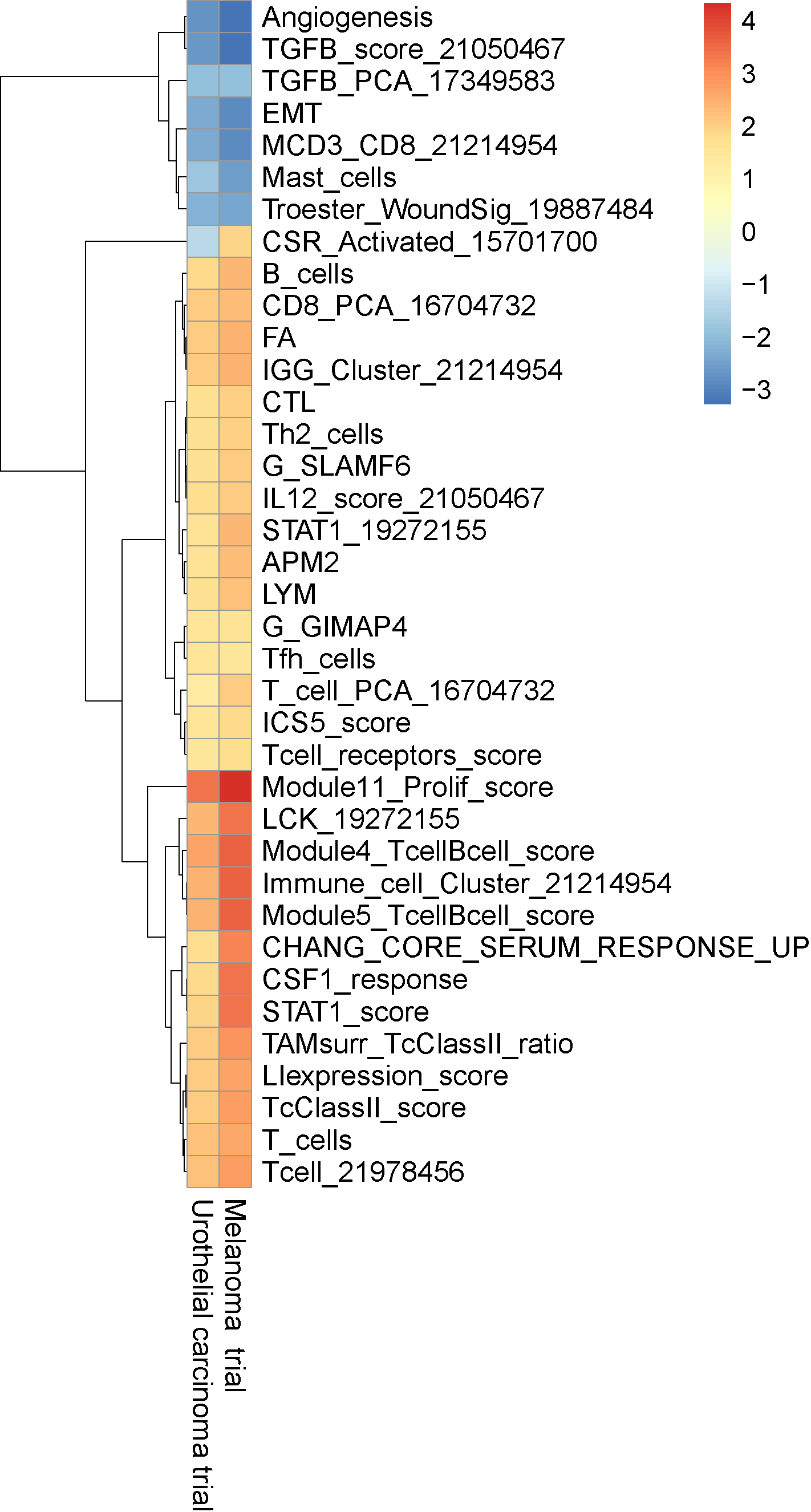


**Figure S3. Gene set enrichment analysis of the identified immunomodulatory signature.** Signaling pathways statistically significant (adjusted p < 0.10) in both two datasets were displayed in the heatmap.

**
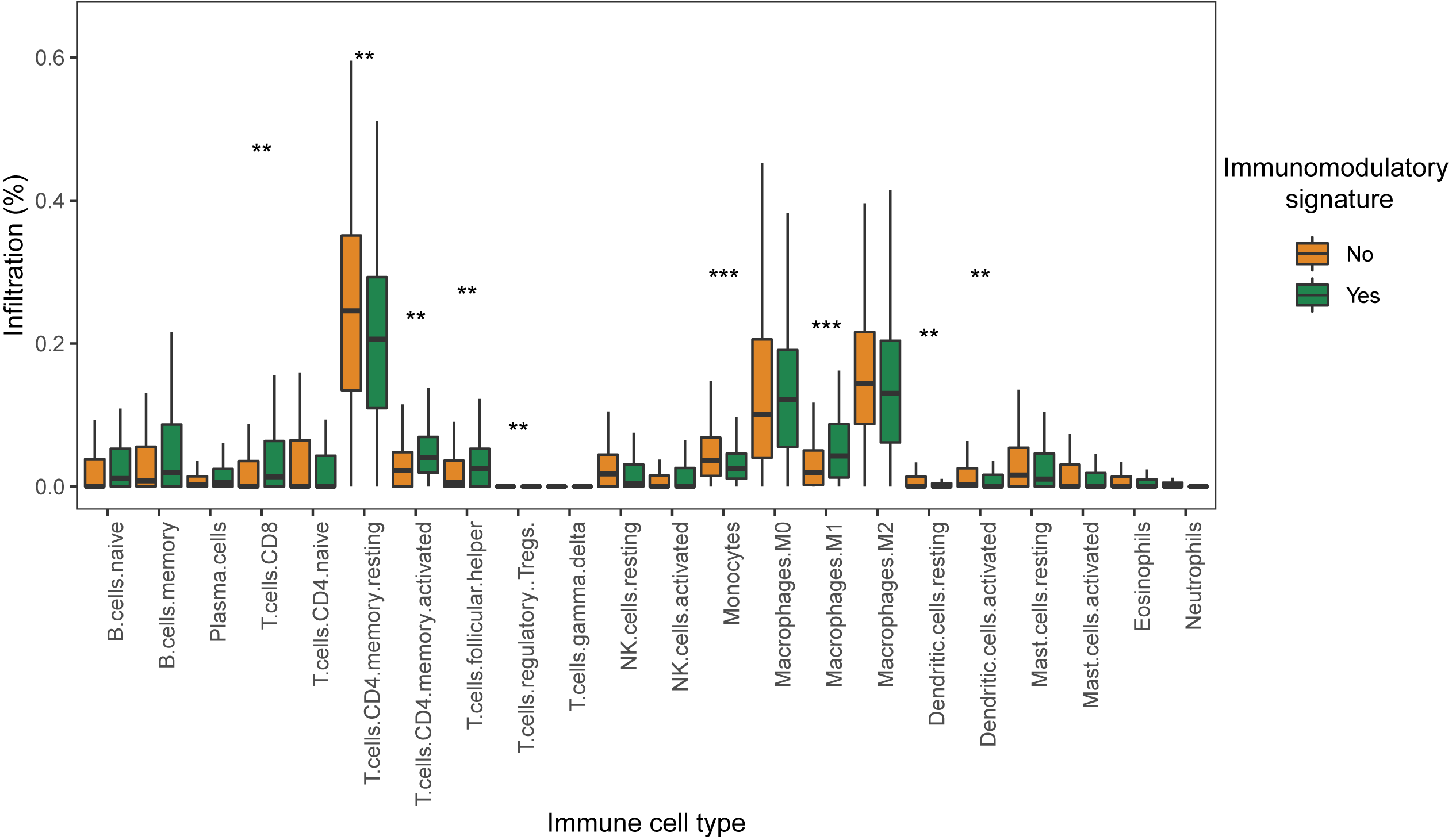
**

**Figure S4. Tumor immune microenvironment signatures unveiled by CIBERTSORT.** The infiltration difference of each immune signature was evaluated by t-test. The p-values were subjected for multiple hypothesis test. (*** represented adjusted p ≤ 0.001 and ** 0.05 < adjusted p < 0.001)

**Table S1.** Data source information

| GEO accession | Samples | Tumor type | Citation |
| --- | --- | --- | --- |
| GSE102130 | 4058 | Glioma | Filbin MG, Tirosh I, Hovestadt V, Shaw ML et al. Developmental and oncogenic programs in H3K27M gliomas dissected by single-cell RNA-seq. Science 2018 Apr 20;360(6386):331-335. |
| GSE103322 | 5902 | Head and neck squamous carcinoma | Puram SV, Tirosh I, Parikh AS, Patel AP et al. Single-Cell Transcriptomic Analysis of Primary and Metastatic Tumor Ecosystems in Head and Neck Cancer. Cell 2017 Dec 14;171(7):1611-1624.e24. |
| GSE108989 | 11138 | Colon cancer | Zhang L, Yu X, Zheng L, Zhang Y et al. Lineage tracking reveals dynamic relationships of T cells in colorectal cancer. Nature 2018 Dec;564(7735):268-272. |
| GSE98638 | 5063 | Liver cancer | Zheng C, Zheng L, Yoo JK, Guo H et al. Landscape of Infiltrating T Cells in Liver Cancer Revealed by Single-Cell Sequencing. Cell 2017 Jun 15;169(7):1342-1356.e16. |
| GSE146771 | 10468 | Colon cancer | Zhang L, Li Z, Skrzypczynska KM, Fang Q et al. Single-Cell Analyses Inform Mechanisms of Myeloid-Targeted Therapies in Colon Cancer. Cell 2020 Apr 16;181(2):442-459.e29. |
| GSE99254 | 12346 | Lung cancer | Guo X, Zhang Y, Zheng L, Zheng C et al. Global characterization of T cells in non-small-cell lung cancer by single-cell sequencing. Nat Med 2018 Jul;24(7):978-985. |
| GSE115978 | 7186 | Melanoma | Jerby-Arnon L, Shah P, Cuoco MS, Rodman C et al. A Cancer Cell Program Promotes T Cell Exclusion and Resistance to Checkpoint Blockade. Cell 2018 Nov 1;175(4):984-997.e24. |
| GSE70630 | 4347 | Glioma | Tirosh I, Venteicher AS, Hebert C, Escalante LE et al. Single-cell RNA-seq supports a developmental hierarchy in human oligodendroglioma. Nature 2016 Nov 10;539(7628):309-313. |
| GSE72056 | 4645 | Melanoma | Tirosh I, Izar B, Prakadan SM, Wadsworth MH 2nd et al. Dissecting the multicellular ecosystem of metastatic melanoma by single-cell RNA-seq. Science 2016 Apr 8;352(6282):189-96. |
| GSE89567 | 6341 | Astrocytoma | Venteicher AS, Tirosh I, Hebert C, Yizhak K et al. Decoupling genetics, lineages, and microenvironment in IDH-mutant gliomas by single-cell RNA-seq. Science 2017 Mar 31;355(6332). |

**Table S2.** Immunomodulatory gene sets.

| Pathway | Gene set |
| --- | --- |
| immunomodulators | *CD80 CD28 ICOSLG PDCD1LG2 CD274 VTCN1 SLAMF7 BTN3A2 BTN3A1 C10orf54 CD276 TNFSF9 TNF TNFSF4 IL1B CXCL9 CXCL10 CCL5 VEGFB CX3CL1 TGFB1 VEGFA CD70 CD40LG IL10 IFNG IL1A IL12A IFNA2 IFNA1 IL4 IL2 IL13 TNFRSF18 PDCD1 HAVCR2 LAG3 CTLA4 TIGIT IL2RA TNFRSF4 CD27 TNFRSF9 ICOS BTLA KIR2DL3 KIR2DL1 TNFRSF14 EDNRB CD40 ADORA2A TLR4 ITGB2 ICAM1 SELP HLA-DRB5 HLA-DQA1 HLA-DQB1 MICA MICB HLA-DQA2 HLA-DQB2 HLA-B HLA-A HLA-C HLA-DRA HLA-DRB1 HLA-DPB1 HLA-DPA1 IDO1 GZMA PRF1 HMGB1 ENTPD1 ARG1* |
| Co-simulator | *CD80 CD28 ICOSLG* |
| Co-inhitor | *PDCD1LG2 CD274 VTCN1 SLAMF7 BTN3A2 BTN3A1 C10orf54 CD276* |
| Ligand | *TNFSF9 TNF TNFSF4 IL1B CXCL9 CXCL10 CCL5 VEGFB CX3CL1 TGFB1 VEGFA CD70 CD40LG IL10 IFNG IL1A IL12A IFNA2 IFNA1 IL4 IL2 IL13* |
| Receptor | *TNFRSF18 PDCD1 HAVCR2 LAG3 CTLA4 TIGIT IL2RA TNFRSF4 CD27 TNFRSF9 ICOS BTLA KIR2DL3 KIR2DL1 TNFRSF14 EDNRB CD40 ADORA2A TLR4* |
| Cell adhesion | *ITGB2 ICAM1 SELP* |
| Antigen presentation | *HLA-DRB5 HLA-DQA1 HLA-DQB1 MICA MICB HLA-DQA2 HLA-DQB2 HLA-B HLA-A HLA-C HLA-DRA HLA-DRB1 HLA-DPB1 HLA-DPA1* |
| Other | *IDO1 GZMA PRF1 HMGB1 ENTPD1 ARG1* |

**Table S3.** Clinical characteristic of the patients with ICB therapy in two clinical trials.

| Characteristic | Urothelial carcinoma clinical trial | | Melanoma clinial trial | |
| --- | --- | --- | --- | --- |
|  |  |  |  |  |
|  | CR or PR (N = 68) | SD or PD (N=230) | CR or PR (N = 47) | SD or PD (N= 72) |
| Sex – no. of patients (%) |  |  |  |  |
| Female | 11 (16.2) | 54 (23.5) | 19 (40.4) | 30 (41.7) |
| Male | 57 (83.8) | 176 (76.5) | 28 (59.6) | 42 (58.3) |
| TMB – mutations |  |  |  |  |
| Median | 448 | 224 | 328 | 203.5 |
| Range | 32-1984 | 0-1408 | 10-6251 | 17-6103 |
| CTL level |  |  |  |  |
| Median | 1.8 | 1.4 | 1.7 | 1.6 |
| Range | -1.9-4.8 | -10.7-5.5 | -3.4-7.0 | -3.3-4.6 |
| *PD-1* expression |  |  |  |  |
| Median | -0.5 | -0.9 | -0.8 | -0.9 |
| Range | -16.6-1.9 | -16.6-3.1 | -7.9-4.8 | -12.4-3.1 |
| *PD-L1* expression |  |  |  |  |
| Median | 2.1 | 1.6 | 2.6 | 2.3 |
| Range | -0.5-5.2 | -2.3-6.1 | -4.4-5.7 | -4.9-7.6 |
| *CTLA-4* expression |  |  |  |  |
| Median | 2.3 | 2.1 | 1.9 | 1.7 |
| Range | -3.0-4.7 | -16.6-5.0 | -5.4-6.9 | -6.1-4.7 |
| TCGA subtype – no. of patients (%) |  |  |  |  |
| I | 23 (33.8) | 84 (36.5) | - | - |
| II | 21 (30.9) | 54 (23.5) | - | - |
| III | 10 (14.7) | 50 (21.7) | - |  |
| IV | 14 (20.6) | 42 (18.3) | - | - |
| Melanoma stage – no. of patients (%) |  |  |  |  |
| M0 | - | - | 0 | 10 (13.9) |
| M1a | - | - | 3 (6.4) | 4 (5.6) |
| M1b | - | - | 6 (12.8) | 8 (11.1) |
| M1c | - | - | 38 (80.9) | 50 (69.4) |
| Overall survival – month |  |  |  |  |
| Median | NA | 8 | NA | 12 |
| Range | 9-24 | 0-24 | 2-56 | 1-44 |

**Table S4.** Immune related signaling gene sets.

| Pathway | Gene set |
| --- | --- |
| Interferon response | *IFNGR1 IFNGR2 STAT1 STAT2 STAT3 JAK1 JAK2 SOCS1 SOCS3 FAS IFNAR1 IFNAR2* |
| Class I MHC presentation | *PSMB8 PSMB9 PSMB10 TAP1 TAP2 CALR CANX PDIA3 TAPBP TAPBPL NLRC5 IRF1 ERAP1 ERAP2 B2M HLA-A HLA-B HLA-C* |
| EMT | *ZEB1 LIX1L VIM AXL MMP2 ANTXR2 C3orf21 FN1 NRP1 TGFBI GALNT5 PPARG HNMT CARD6 RBPMS TNFRSF21 TMEM45B MPP7 SSH3 MTAC2D1 MUC1 EPPK1 SHROOM3 EPN3 PRSS22 AP1M2 SH3YL1 KLC3 SERINC2 EVPL FXYD3 CLDN4 CRB3 LRRC54 MAPK13 GALNT3 STAP2 DSP ELMO3 KRTCAP3 MAL2 F11R GPR110 GPR56 KRT19 GRHL1 BSPRY C1orf116 S100A14 SPINT2 ANKRD22 ST14 GRHL2 PRR5 TJP3 TACSTD2 CDH3 C1orf172 CDS1 MPZL2 INADL RBM35A TMC4 ITGB6 TMEM125 EPHA1 ENPP5 EPB41L5 ERBB3 RAB25 PRSS8 TMEM30B CLDN7 TACSTD1 SCNN1A CDH1 CHD2* |
| BER | *UNG SMUG1 MBD4 TDG OGG1 MUTYH NTHL1 MPG NEIL1 NEIL2 NEIL3 APEX1 APEX2 LIG3 XRCC1 PNKP APLF* |
| PARP | *PARP1 PARP2 PARP3* |
| MMR | *MSH2 MSH3 MSH6 MSH4 MSH5 MLH1 MLH3 PMS2 PMS1 PMS2L3* |
| NER | *XPC RAD23B CETN2 RAD23A XPA DDB1 DDB2 RPA1 RPA2 RPA3 TFIIH ERCC3 ERCC2 GTF2H1 GTF2H2 GTF2H3 GTF2H4 GTF2H5 CDK7 CCNH MNAT1 ERCC5 ERCC1 ERCC4 LIG1 ERCC8 ERCC6 UVSSA XAB2 MMS19* |
| HR | *RAD51 RAD51B RAD51D DMC1 XRCC2 XRCC3 RAD52 RAD54L RAD54B BRCA1 SHFM1 RAD50 MRE11A NBN RBBP8 MUS81 EME1 EME2 GIYD1 GIYD2 GEN1* |
| FA | *FANCA FANCB FANCC FANCD2 FANCE FANCF FANCG FANCI FANCL FANCM BRCA2 BRIP1 PALB2 RAD51C BTBD12 FAAP20 FAAP24* |
| NHEJ | *XRCC6 XRCC5 XRCC4 PRKDC LIG4 DCLRE1C NHEJ1* |
| DNA polymerases | *POLB POLG POLD1 POLE POLH POLI POLQ POLK POLL POLM POLN REV3L MAD2L2 REV1L* |
| PD1 | *PDCD1* |
| T cell dysfunction | *KCNMA1 ADAM19 NDST3 VOPP1 CD5 IL2RB RORA GPR155 BTN3A1 ICOS TREML1 CD274 ZNFX1 NLRC5 POU2F2 ADGRG5 ELMO1 SPN NFATC3 TGFB1 ANKRD29 PNMT STARD3 ARID3B KCNA5 LRMP FAM65B COQ10A SERPINB9 NDST4 DNAJC30 TMPRSS3 USH1G GUCA1A VSNL1 COL11A2 SFN NAV2 ADAMTS17 WNT10A XCL1 KHNYN MAL WFS1 DIDO1 NUB1 LIME1 TMEM130 SOX10 CCDC43 TRIT1 HDAC2 ATP5C1 PAPSS1 PGM3 COPB2 PHGDH LMBRD2 DXO HSDL2 PEX13 NDUFS2 COPG1 TIPRL ST3GAL6 SESTD1 DDX59 PPP3CA SNRNP48 SEC23A OSTC AXIN2 CACNA2D2 BMP4 PDE1A GRK2 KCNA3 KLRB1 SPOCK2 CD6 PVRIG LY9 FCRL5 PIM2 RHOF ZBTB32 IL21R ZBTB7A LRRC15 AMPD3* |
| CTL | *CD8A CD8B GZMA GZMB PRF1* |
| aDC | *CCL1 EBI3 INDO LAMP3 OAS3* |
| Angiogenesis | *CDH5 ELTD1 CLEC14A LDB2 ECSCR MYCT1 RHOJ VWF TIE1 KDR ESAM PTPRB GPR116 SPARCL1 EMCN ROBO4 ENG TEK S1PR1 A2M JAM2 COL15A1 PECAM1 CALCRL CLEC3B PLVAP RGS5 LRRC32 EBF1 ADCY4 ACVRL1 GPR124 APLNR TM4SF18 GNG11 CNRIP1 ZNF423 GIMAP8 PDGFD ITGA9 EDNRB* |
| APM1 | *HLA-A HLA-B HLA-C TAP1 TAP2 TAPBP B2M HLA.A HLA.B HLA.C* |
| APM2 | *HLA-DMA HLA-DMB HLA-DOA HLA-DOB HLA-DPA1 HLA-DPB1 HLA-DPB2 HLA-DQA1 HLA-DQA2 HLA-DQB1 HLA-DQB2 HLA-DRA HLA-DRB1 HLA-DRB5 HLA-DRB6 HLA.DMA HLA.DMB HLA.DOA HLA.DOB HLA.DPA1 HLA.DPB1 HLA.DPB2 HLA.DQA1 HLA.DQA2 HLA.DQB1 HLA.DQB2 HLA.DRA HLA.DRB1 HLA.DRB5 HLA.DRB6* |
| B_cell_PCA_16704732 | *SLC22A3 IGKC FGD2 SIDT2 CACNA2D2 USP6NL CPSF2 UBR5 NIPAL4 WEE1 PMAIP1 SLC2A5 BEND4 SP140 GLDC GSTZ1 EML6 FCRLA LRMP BTK HLA-DMB SAV1 PLEKHF2 STAG3 HHEX LINC00926 WDR11 SSU72 CD19 SYNGR2 ZNF532 TRIM56 MEF2C INPPL1 NFKBIE JUP EGR1 FCRL2 TBC1D1 ANKH TRIO RGS13 SSPN ATG4A C11orf24 PRKCE RALGPS2 AIM2 PIK3AP1 BACE2 COPS3 CD22 FCRL5 PHF16 POLD4 TSPAN33 EPHX1 PEA15 CNR2 IFNGR2 TSPAN31 PCCA ANXA4 RAP1GAP2 MYBL2 CD72 CIITA PMEPA1 CR1 ITPR1 IFI27 HLA-DOA CDKN2A ZNF207 SYT17 EVI5L TUBB6 DDR1 COL14A1 CHD7 APOBEC3B FAM129C SCRN1 TAP2 MARCKS SNX10 CCNG2 CYBB BRD4 PLEKHO1 RNASE6 STAP1 ATP6V0A1 VPS53 RFX3 MOB3B KDM4B CORO1C TCL1A RAB30 SEMA4B DENND5B ARHGEF3 UVRAG TSPAN3 PARP14 CTSZ HLA-DPB1 F5 PPAPDC1B ZYG11B GSAP BLK CLIC4 HUWE1 NAA50 DAPP1 FIG4 SRGAP2 FCGR2B BCL7A GUSBP11 OSBPL10 CD200 RHOH SH3BP5 CEBPB GNA12 PALM2-AKAP2 NKG7 IRF8 TNFRSF17 CYB561A3 ADK CORO2B CD86 WDR83OS STX7 IGJ CD1D HLA-DPA1 KLHL14 RALGAPB HLA-DQB1 HCG18 RCSD1 PSEN2 MZB1 HRK TNFRSF18 IL4R MTPN DRAM2 TEAD2 MTSS1 SMAGP NCF4 RBMS3 SKAP2 HSPA5 HIST1H2BK CR2 ODC1 LHFPL2 TCTN1 ADRBK2 DMXL1 KYNU PIK3C2B FUBP1 HLA-DRA CCDC25 BMF SETBP1 SAMD9 MGME1 FCRL1 PUS10 CD180 RNF141 CD79A LY86 TNS3 NUP88 TLR7 HVCN1 ZNF154 BTLA RFX5 ZCCHC7 SNX29P2 CYTH1 BTNL9 CD79B GYLTL1B CD38 SYVN1 IFIT3 BLNK HERPUD1 DTX1 RHOBTB2 CXCR5 KLF1 VPREB3 CHERP SLC2A1 E2F5 HECW2 WDR34 TLR10 LYN IGHG1 CCDC50 KAZN PLCG2 MYO1E CORO1A POU2F2 AFF3 GM2A POU2AF1 FNBP4 UROS BIRC3 WIPF1 TPD52 IGL MS4A1 BCL11A SMAD3 ADAM28 DNAJC10 STAT6 HLA-DQB2 IGLL1 ABCA1 MAP3K8 BSG LETMD1 SMC6 SPIB NR1H2 SEL1L3 FOXP1 TOP2A HLA-DRB1 IRF4 LGALS9 UBE2J1 43160 ACTA2 SHMT2 RRAS2 SYK GCNT1 SYPL1 SUN2 MMP11 SNX2 HLA-DQA1 AMFR PRICKLE1 TTC7A SLC15A2 CD74 TFEB CD83 ARHGAP24 FBXO41 SWAP70 BANK1 SERPINA1 L3MBTL4 PRCP DNAH11 GRAMD1C GUCD1 TLR1 KIAA0226L HLA.DMB HLA.DOA HLA.DPB1 PALM2.AKAP2 HLA.DPA1 HLA.DQB1 HLA.DRA HLA.DQB2 HLA.DRB1 HLA.DQA1* |
| B_cells | *ABCB4 BACH2 BCL11A BLK BLNK CCR9 CD19 CD72 COCH CR2 DTNB FCRL2 GLDC GNG7 HLA-DOB HLA-DQA1 IGHA1 IGHG1 IGHM IGKC IGL KIAA0125 MEF2C MICAL3 MS4A1 OSBPL10 PNOC QRSL1 SCN3A SLC15A2 SPIB TCL1A TNFRSF17 HLA.DOB HLA.DQA1* |
| Bcell_21978456 | *IGHA1 IGHG1 IGHM IGH IGHA2 IGHD IGHG3 IGHG4 IGHV4-31 IGHV1-69 IGLJ3 IGHV3-23 IGK IGHG2 IGKC GUSBP11 IGLC1 IGLV1-44 CYAT1 IGLV NA IGKV1D-13 IGLL3P IGLL5 SCFV IGHV4.31 IGHV1.69 IGHV3.23 IGLV1.44 IGKV1D.13* |
| Bcell_mg_IGJ | *IGL IGHM IGHG3 IGHV1-69 LOC652128 IGKC IGLJ3 IGJ IL8 NTN2L CTA-246H3.1 IGKV1D-13 IFI6 IGHA1 IGKV1OR2-108 IGKV1OR15-118 IGHV1.69 CTA.246H3.1 IGKV1D.13 IGKV1OR2.108 IGKV1OR15.118* |
| Bcell_receptors_score | *CD19 CD1C CD1D CD20 CD200 CD22 CD24 CD38 CD72 CD74 CD79A CD79B CD83 CD86 FCGR2B* |
| Buck14_score | *CXCL13 CLIC5 RGS4 RPS28 RFX7 EXOC7 HAPLN1 ZNF3 SSX3 HRBL PRRG3 ABO PRTN3 MATN1* |
| CD103neg_mean_25446897 | *ABCC3 APOE C1QA C1QB C1QC C5AR1 CYP4F18/CYP4F3A LYVE1 MERTK MRC1 MS4A7 SIGLEC1 STAB1 TLR7 TMEM119 TMEM37 TREM2* |
| CD103pos_mean_25446897 | *BATF3 BTLA CCR7 CLEC9A FLT3 IRF8 KIT MYCL1 XCR1 ZBTB46* |
| CD103pos_neg_ratio_25446897 | *CD103pos_mean_25446897 CD103neg_mean_25446897* |
| CD68 | *CD68* |
| CD8_CD68_ratio | *CD8A CD68* |
| CD8_PCA_16704732 | *DUSP2 GPR56 CST7 FCGBP S100B ADRB2 KLRC4 CCL5 IL2RB GNLY KLRC1 CD8A C1orf21 CCL4L1 TBX21 TSPAN32 KLRG1 PRF1 GZMH* |
| CD8_T_cells | *ABT1 AES APBA2 ARHGAP8 C12orf47 C19orf6 C4orf15 CAMLG CD8A CD8B CDKN2AIP DNAJB1 FLT3LG GADD45A GZMM KLF9 LEPROTL1 LIME1 MYST3 PF4 PPP1R2 PRF1 PRR5 RBM3 SF1 SFRS7 SLC16A7 TBCC THUMPD1 TMC6 TSC22D3 VAMP2 ZEB1 ZFP36L2 ZNF22 ZNF609 ZNF91* |
| CD8A | *CD8A* |
| CHANG_CORE_SERUM_RESPONSE_UP | *CEP78 LSM3 LRRC40 STK17A RPN1 JUNB NUP85 FLNC HMGN2 RPP40 UQCR10 AIMP2 CHEK1 VTA1 EXOSC8 CENPO PNO1 SLC16A1 WDR77 UBE2J1 NOP16 NUDT1 SMC2 SLC25A5 NUPL1 DLEU2 PDAP1 CCBL2 COX17 BCCIP PLG RGS8 SNRPC PLK4 NUTF2 LSM4 SMS EBNA1BP2 C13orf27 VDAC1 PSMD14 MYCBP SMURF2 GNG11 F3 IL7R BRIP1 HNRNPA2B1 DCK ALKBH7 HN1L MSN TPM1 HYLS1 HAUS1 NUP93 SNRPE ITGA6 CENPN C11orf24 GGH PFKP FARSA EIF2AK1 CENPW TUBA4A TRA2B UMPS MRTO4 NUDT15 PGM2 DBNDD1 SNRPB MNAT1 NUP35 TCEB1 HSPB11 C19orf48 ID3 IPO4 FARSB EIF4G1 SKA1 MFSD11 PLAUR MARVELD2 MCM3 DHFR RNF41 ID2 H2AFZ CDK2 NCLN ZWILCH DYNLT1 C16orf61 SLC25A40 RHOC CCT5 PDIA4 SNRPA RBM14 PDLIM7 PITPNC1 TPM3 CORO1C ERLIN1 PAICS TPRKB SKA2 MYBL1 SH3BP5L BRCA2 SAR1A POLR3K MRPS28 NUP107 TUBG1 PNN FAM167A RFC3 MYL6 MCM7 MAGOHB FAM89B TOMM40 CDCA4 MT3 MTHFD1 PSMD12 MYBL2 CKLF NRIP3 EZR C12orf24 GPLD1 SRM RAB3B NLN MT1F TNFRSF12A TPI1 HAS2 APOO FBXO41 MRPL37 GSTCD SDC1 WDR54 RNF138 APITD1 RMND5B ENO1 MAP3K8 TMEM130 SNX17 KRR1 TAGLN PA2G4 RUVBL1 SNRPD1 LOXL2 POLE2 MAPRE1 IMP4 EMP2 PSMD2 MET IFRD2 LMNB2 PLOD2 NCEH1 NME1 STRA13 ACTL6A DLEU1 SNRPA1 CBX1 LYAR PTPLB PFN1 CENPJ COTL1 SPRYD7 USPL1 MRPL12 ADAMTS1 GLRX3 WSB2 MRPS16 DCLRE1B MKKS C3orf26 CPEB4 SPAG17 MLF1IP UAP1 COQ2 WDHD1 DCBLD2 KIAA0090 SAR1B PSMA7 PSMC3 COPS6 DUT PPIH PHF19 TPM2 MCTS1 EIF4EBP1 HNRNPR* |
| Chemokine12_score | *CCL2 CCL3 CCL4 CCL5 CCL8 CCL18 CCL19 CCL21 CXCL9 CXCL10 CXCL11 CXCL13* |
| CSF1_response | *CORO1A MNDA CCRL2 SLC7A7 HLA-DMA FYB RNASE6 TLR2 CTSC LILRB4 PSCDBP CTSS RASSF4 MSN CYBB LAPTM5 DOCK2 FCGR1A EVI2B ADCY7 CD48 ARHGAP15 ARRB2 SYK BTK TNFAIP3 FCGR2A VSIG4 FPRL2 IL10RA IFI16 ITGB2 IL7R TBXAS1 FMNL1 FLI1 RASSF2 LYZ CD163 CD97 CCL2 FCGR2B MERTK CD84 CD53 CD86 HMHA1 CTSL EVI2A TNFRSF1B CXCR4 LCP1 SAMHD1 CPVL HLA-DRB1 C13orf18 GIMAP4 SAMSN1 PLCG2 OSBPL3 CD8A RUNX3 FCGR3A AMPD3 MYO1F CECR1 LYN MPP1 LRMP FGL2 NCKAP1L HCLS1 SELL CASP1 SELPLG CD33 GPNMB NCF2 FNBP1 IL18 B2M SP140 FCER1G LCP2 LY86 LAIR1 IFI30 TNFSF13B LST1 FGR NPL PLEK CCL5 PTPRC GNPTAB SLC1A3 HCK NPC2 C3AR1 PIK3CG DAPK1 ALOX5AP CSF1R CUGBP2 APOE APOC1 CD52 LHFPL2 C1orf54 IKZF1 SH2B3 WIPF1 HLA.DMA HLA.DRB1* |
| CSR_Activated_15701700 | *ACAS2 ACINUS ADAMTS1 ADD3 AIP1 ALEX3 APOD APP ARHC ARHGAP12 ARHGEF3 ATP6V0A1 AXIN2 BAF53A BBC3 BCCIP BCL6 BF BHC80 BHLHB2 BM039 BOCT BRCA2 BRIP1 C11ORF14 C11ORF24 C13ORF1 C1ORF33 C1S C20ORF108 C5ORF4 C6ORF55 C8ORF13 CAMLG CBX1 CCNG2 CCNL2 CCT5 CDC14B CDCA4 CDK2 CDKN1A CDKN1C CENPJ CGI-121 CHEK1 CKLF CL640 CLCN6 CLIC2 CNTNAP1 COPS6 CORO1C COTL1 COX17 CPR8 CRTAP CSF1 CST3 CTNS CTSF CYP27A1 DC13 DCK DCLRE1B DEPP DHFR DHRS6 DKFZP434B103 DKFZP434L142 DKFZP434O1427 DKFZP586A0522 DKFZP727G051 DKFZP761L1417 DKFZP762H185 DLEU1 DLEU2 DNM1 DPP7 DPYSL2 DUSP22 DUT EBNA1BP2 EEF1E1 EIF4EBP1 EIF4G1 EMP2 ENIGMA ENO1 EPHB1 ERP70 ESDN F10 F3 FABP3 FACL3 FADS1 FADS2 FARSL FCGRT FDPS FLJ10036 FLJ10292 FLJ10407 FLJ10618 FLJ10849 FLJ10948 FLJ10983 FLJ11286 FLJ12643 FLJ12953 FLJ14525 FLJ20059 FLJ20154 FLJ20331 FLJ21016 FLJ21986 FLJ23462 FLJ23468 FLJ30532 FLJ30574 FLJ31033 FLJ32731 FLJ32915 FLJ90754 FLJ90798 FLNC FRSB FYCO1 GABARAPL1 GABBR1 GATM GG2-1 GGH GLS GNG11 GPLD1 GPR124 GSN H2AFZ H2AV HAS2 HDAC5 HECA HMGB1 HMGCS1 HMGN2 HN1L HNRPA2B1 HNRPR HRB2 HRI HSPC111 HSU79274 ID2 ID3 IDI1 IFRD2 IGBP1 IL1R1 IL6ST IL7R IMP4 INSIG1 IPO4 ITGA6 ITPKB JAG1 JTV1 KAI1 KEO4 KIAA0090 KIAA0095 KIAA0323 KIAA0342 KIAA0367 KIAA0874 KIAA1036 KIAA1109 KIAA1228 KIAA1268 KIAA1305 KIAA1363 KIAA1536 KIAA1554 KIAA1720 KIAA1946 KIT KLHL5 LDB1 LDLR LIPA LMNB2 LOC115106 LOC115294 LOC129401 LOC153222 LOC169611 LOC201562 LOC201895 LOC221810 LOC253263 LOC284018 LOC284436 LOC285362 LOC339924 LOC51128 LOC51279 LOC51668 LOC56757 LOC56902 LOC56926 LOC93081 LOXL2 LPIN1 LRIG2 LRP1 LSM3 LSM4 LSS LUM LYAR MAF MAN1A1 MAP3K8 MAPRE1 MBP MCM3 MCM7 MCP MCT-1 MEF2D MEP50 MET MFGE8 MGC10200 MGC10500 MGC10974 MGC11266 MGC13047 MGC13170 MGC14480 MGC15429 MGC17330 MGC3101 MGC39820 MGC4170 MGC4308 MGC4825 MIG-6 MKKS MNAT1 MRF2 MRPL12 MRPL37 MRPS16 MRPS28 MSN MT1F MT3 MTH2 MTHFD1 MVD MXI1 MYBL1 MYBL2 MYCBP MYL6 NCOA3 NIFU NLN NME1 NOLA2 NUDT1 NUP107 NUPL1 NUTF2 OIP2 OSBPL8 P8 PA26 PA2G4 PAICS PBXIP1 PCNT1 PCSK7 PDAP1 PDK2 PFKP PFN1 PGCP PHACS PITPNC1 PKIG PLA2R1 PLAUR PLD3 PLG PLOD2 PLU-1 PLXNB1 PNN POLE2 POLR3K POR PPIH PROL2 PSMA7 PSMC3 PSMD12 PSMD14 PSMD2 PTPRU PTS RAB11-FIP2 RARRES3 RBM14 RBMX REV3L RFC3 RNASEH2A RNASEP1 RNF138 RNF41 RPN1 RRM2B RUVBL1 SATB1 SBP1 SC4MOL SCD SDC1 SDFR1 SELENBP1 SEMACAP3 SERPING1 SES2 SFRS10 SFRS2 SFTPB SLC16A1 SLC25A5 SLC35E2 SLC40A1 SLC5A3 SLPI SMC2L1 SMS SMURF2 SNRP70 SNRPA SNRPA1 SNRPB SNRPC SNRPD1 SNRPE SQLE SQRDL SRM SSR3 SSSCA1 STK17A STK18 SVIL TAGLN TBRG1 TCEB1 TCTEL1 TFPI2 TIM50L TIMP2 TNFAIP2 TNFRSF12A TNFSF12 TNXB TOMM40 TP53INP1 TPI1 TPM1 TPM2 TRIM22 TSC22 TUBA1 TUBG1 TXNL2 UAP1 UBE2J1 UMPK UMPS VAMP4 VDAC1 VIL2 WBP2 WDHD1 WSB1 WSB2 WTAP ZFP106 ZNF151 ZNF219 ZNF36 ZNF83 CGI.121 GG2.1 MCT.1 MIG.6 PLU.1 RAB11.FIP2* |
| CTLA4 | *CTLA4* |
| CTLA4_data | *CTLA4* |
| Cytotoxic_cells | *APBA2 APOL3 CTSW DUSP2 GNLY GZMA GZMH KLRB1 KLRD1 KLRF1 KLRK1 NKG7 RORA RUNX3 SIGIRR WHAMMP3 ZBTB16* |
| DAP12_data | *TYROBP* |
| DC | *CCL13 CCL17 CCL22 CD209 HSD11B1 NPR1 PPFIBP2* |
| Eosinophils | *ABHD2 ACACB C9orf156 CAT CCR3 CLC CYSLTR2 EMR1 EPN2 GALC GPR44 HES1 HIST1H1C HRH4 IGSF2 IL5RA KBTBD11 KCNH2 LRP5L MYO15B RCOR3 RNASE2 RRP12 SIAH1 SMPD3 SYNJ1 TGIF1 THBS1 THBS4 TIPARP TKTL1* |
| G_CD3E | *CD3E CD3D IL10RA AMICA1 CD3G* |
| G_CYTH4 | *CYTH4 NCF4 RAC2 IL2RB CSF2RB* |
| G_GIMAP4 | *GIMAP4 GIMAP5 GIMAP6 GIMAP8 GIMAP7 GIMAP1* |
| G_HLA-DPA1 | *HLA-DPA1 HLA-DRA HLA-DPB1 HLA-DRB1 HLA-DMB HLA-DMA HLA-DOA HLA-DQA1 HLA-DQB1 HLA-DRB5 HLA.DPA1 HLA.DRA HLA.DPB1 HLA.DRB1 HLA.DMB HLA.DMA HLA.DOA HLA.DQA1 HLA.DQB1 HLA.DRB5* |
| G_LILRB4 | *LILRB4 LILRB2 LAIR1 LILRB1 LILRB3 LILRA6 OSCAR* |
| G_SIGLEC9 | *SIGLEC9 SIGLEC7 FPR3 SIGLEC5 CD33 SIGLEC10 SIGLEC14 FPR1* |
| G_SLAMF6 | *SLAMF6 SLAMF7 CD48 LY9 SLAMF1 ARHGAP30 SLAMF8* |
| GP11_Immune_IFN | *Minterferon_Cluster_21214954 ZHANG_INTERFERON_RESPONSE* |
| GP2_ImmuneTcellBcell_score | *KEGG_HEMATOPOEITIC_CELL_LINEAGE REACTOME_SIGNALING_IN_IMMUNE_SYSTEM* |
| GRANS_PCA_16704732 | *SPTBN2 SLPI CMTM6 SLC9A7 SLC25A37 GABARAP QPCT PARPBP AOX1 PPM1A RER1 LAMP2 SGCE OSBP2 FAXDC2 TGFA LRRK2 SPRTN NFIA MOG BNIP3L CDA C5orf45 AGO4 CAMSAP2 TNFRSF10A CYP4F3 ELF4 RAB2B TST TBC1D10C IL18RAP BST2 FIS1 SERPINE1 ATP6V0C SLC31A2 ARRB2 ZNF598 RTN3 OAZ1 GYPB USP47 CHI3L1 EMR2 C9orf78 BACH1 SLC7A7 ALOX5AP HLA-E TNFRSF10C TSPAN7 MX2 CLEC2D PIP MECOM SELENBP1 TSPAN2 GLIPR1 SMOX CCL4L1 ALPL FCGRT BIN2 ANAPC16 SDCBP DLX4 DHRS7 FTL C16orf72 CPQ SLC7A5P1 PYGL ICAM3 SLC45A4 C9orf40 XK STX3 PSMB3 DEFA1 DYSF CDK18 GSPT1 NDE1 CECR6 SLC15A4 TAS2R38 PLEKHG3 DUSP6 ST14 S100A11 FAM104A PPM1D RAC1 FKBP8 RGS20 GRINA IDH2 IL13RA1 HCAR2 PLEK2 BCL2L1 SLC25A44 ANKIB1 GNB2 HSPA1A TIAM2 CSF3R CD46 NFE2 NOV CHI3L2 S100P TALDO1 NAGA SAP30 C3AR1 TM4SF1 TMCC2 VNN2 RIPK2 PDK1 NRBF2 ACVR1B SYNE2 ZNF185 CXCR2 CAMP PSMB9 SNCA IKBKG WASH5P DHRS9 CHST15 MKRN1 KCTD1 CD1B TIGD3 RPS2 LYZ CREB5 RPS6KA5 LGALS3BP TMCC3 DIRAS3 CSF2RB DNAJC8 OXCT1 CTSC IQSEC1 PRC1 IL1R2 ANXA5 SETD5-AS1 EMC3 ERRFI1 CITED2 YBX3 IMPA2 LDHB PLBD1 ALDH2 CD48 GUK1 NINJ2 MPP1 LRRN3 RFXANK RASSF2 PICALM SH3GLB1 CDC34 NCOA4 ACOX1 MYD88 SLC22A4 RNASE2 SEC14L1 ACSL1 PLXNC1 UBN1 FAM129A RXRA C20orf194 AQP9 CDIPT TMEM140 GLUL NXPH3 MAP2K3 DFNA5 FMOD CPVL HLX TM9SF2 OLIG1 JAZF1 MARVELD2 CCDC91 DCAF12 BEST1 DNASE1 ANK2 NPL ITM2B ST6GALNAC2 TLR8 ABHD5 ABTB1 GSN IFIT2 MSRA GATA6 TECPR2 UBE2B TSTD2 MIIP MB FGL2 GRN ARAP3 LGALS3 ZIC2 HBG1 SLA RGS2 NAMPT STX6 UBE2L6 CA2 SERPING1 VMP1 MFSD1 PCK1 LOC644462 MYCN PIM1 SPOPL SIGLEC5 PIGS LIMK2 TMEM259 EVI2B PAK1 CREG1 UBXN6 ART4 EIF2AK1 ALAS2 LMO2 CNPPD1 APOC2 FCGR3A PI3 PGD TBX19 SIAH2 IFITM2 COL16A1 MME RAB9A SNX27 CHMP2A CD302 FAM214B KLF1 SQRDL AOC1 SOD2 CMBL GMPR ATP6V1B2 NBPF12 LYN NCF2 MXD1 ATP6V0B BCL6 IL1RN KLHL12 MET CHP1 ZNF599 SOAT1 SEC24A GCA PPP1R18 FPR1 RBM7 DCLRE1C CD40 DOCK5 CNGB3 ADM RAB10 HPCA SH3BGRL3 TPPP2 H3F3A MAPK1 MAD2L1BP SLC25A39 TSPAN5 CBX4 TMCC1 MMP9 TTR CELF2 KRT8 NPRL3 RFX1 MCTP2 SRGN TIMM21 SUMF1 EPB42 LINGO2 FRG1B LECT2 CTSS MAP1A INHBB SERPINB7 WT1 LILRA2 PRSS50 MOB3A RWDD3 ZDHHC18 PPP1R3D CETN3 KCNJ2 PPM1F NR1H4 RNPEP MNDA 43160 MYADM PCSK1 TFE3 RAD50 MEGF9 PGAM1 C17orf103 GPX1 TLR1 XPO6 HLA.E SETD5.AS1* |
| HER2_Immune_PCA_18006808 | *AOC1 ACTN1 ACVR1 ACVRL1 ADAM8 AGER AIF1 ALDH1A1 ALDOB AKR1B1 ALOX5AP AMPD1 AMPD3 BIN1 ANG ANGPT2 ANK1 AOAH APBA2 BIRC3 APOC4 APOE FASLG RHOG ARHGAP1 ARRB2 ATP2A1 B2M BCL2A1 TNFRSF17 BDH1 CXCR5 BNIP3 DST BTK BTN1A1 SERPING1 C1QA C1QB C1R C1S C2 C3 C3AR1 FMNL1 CAPN2 CASP1 CASP4 RUNX1 RUNX3 CCND2 CCND3 CD1D CD1E CD2 CD3D CD3E CD3G CD247 CD6 CD7 CD8A CD8B CD14 CD19 MS4A1 CD22 CD27 CD28 CD80 CD86 CD33 CD37 CD38 ENTPD1 CD40 CD47 CD48 CD53 CD69 CD72 CD74 CD79A CD79B CD97 CDKN2D CDS1 CD52 CTSC CGB CHIT1 CLIC2 CCR1 CCR5 CCR6 CCR7 CMKLR1 COL4A1 COL4A3 COL10A1 COL11A1 CSF1R CSF2RA CSF2RB CSK CSNK1A1 CSRP1 CST3 CTLA4 CTSG CTSH CTSS CTSW CYBA CYBB CYLD CYP11A1 DGKA DBN1 DNASE1L3 DNMT1 DOCK2 DOK1 DRP2 DTNB GPR183 EFNA1 EFNB1 EMP3 ENPEP EPHB2 EPOR EVC EVI2B F13B PTK2B FAU FCER1G FCGRT FCN1 FGR FHL2 FOXO1 FLI1 FLT3LG FOLR2 FPR1 FUCA1 FUT4 FYB FYN G6PC GBP1 GBP2 GFI1 GFPT1 GNA11 GNG7 CXCR3 GPR15 GPR18 GPR27 GPR35 GRK5 GRK6 GRN NR3C1 GRP GSPT1 GUCY1B3 GYPC GZMH GZMA GZMB GZMK GZMM HBBP1 HCK HCLS1 NCKAP1L CFHR2 HHEX HLA-A HLA-B HLA-DMA HLA-DMB HLA-DOA HLA-DOB HLA-DPA1 HLA-DPB1 HLA-DQA1 HLA-DQB1 HLA-DRA HLA-DRB1 HLA-E HLA-G HNRNPH2 HSD11B1 HSPA1B HTR5A ICAM2 ICAM3 IRF8 IFI35 SP110 IFNAR2 IFNG IGHA1 IGHD IGHM IGJ IGLC1 IGLL1 IL2RB IL2RG IL6R IL7 IL7R CXCR2 IL9R IL10RA IL15 IL15RA IL16 TNFRSF9 IDO1 CXCL10 INPP5A INPP5D IRF1 IRF4 IRF5 IRF6 IRF7 ISG20 ITGA4 ITGAM ITGB2 ITGB7 ITK JAK2 KIF2A KIR2DL4 KLRB1 KLRC1 KLRC3 KLRD1 KRTAP5-9 LAG3 LAIR1 LAIR2 LAMC1 LCK LCP1 LCP2 LEPR LGALS2 LGALS9 LMO2 LOXL2 LRMP LRCH4 LSP1 LTB LY9 CD180 LY75 LYL1 LYN SH2D1A MAGEB4 MAL MAN2B1 MAP1B MAP4 MATK MBP MEF2C MFNG MICB CXCL9 MNDA MPP1 MRC1 MYO1F MTX1 MYD88 MYH10 MYO1D MYO6 MYO7A NUBP1 NELL2 NF1 NFATC3 NFKBIA NFKBIE NKG7 NPAT OAZ1 OPHN1 P2RX5 P2RX7 PARN PAX1 PAX5 PFKFB4 PGM3 ABCB1 PHKA1 SERPINB9 PIM1 PIK3CD PIP4K2A PKP2 PLCG2 PLEK PLOD2 PLSCR1 PNOC PNLIPRP1 POU2AF1 POU2F1 PPIC PPP1CB PPP3CC PRCP PRF1 PRKCB PRKCQ MAPK6 MAPK9 PSAP PSMB8 PSMB9 PSMB10 PTAFR PTGDS PTGER4 PTPN1 PTPN6 PTPN7 PTPN12 PTPRC PTPRCAP PTPRF ABCD4 PYCR1 PYGB RAB2A RAB3IL1 RAC1 RAC2 RARRES3 RASA2 RELB RFX5 RGS10 RGS13 RNASE6 RPL23A RPL27A RPLP2 RPS6KA3 RPS28 RTN2 MSMO1 SCP2 CCL2 CCL5 CCL8 CCL13 CCL18 CCL19 CCL23 CXCL11 XCL1 SDC2 SELL SELP SELPLG SEPP1 SLA SLAMF1 SLC1A3 SLC5A5 SNRPC SOD2 SOX4 SP4 SPI1 SPIB SPP1 SPRR1A TRIM21 STAT1 STAT4 STAT5A STK10 SYK TAP1 TAP2 TBXA2R TCF7 TCN2 TRA DYNLT3 TGFB1I1 THBS1 THBS2 TIAM1 TIMP3 TJP1 TLR1 TLR2 TNF TNNC2 TNFAIP3 TNFRSF1B TPM1 TPMT NR2C2 TRAF1 PHLDA2 TTC3 TYROBP UBA7 VAV1 VCAM1 VCL VEGFA VLDLR WARS WAS WIPF1 LAT2 XPNPEP2 ZAP70 TRIM25 LAPTM5 PXDN CXCR4 LST1 NUP214 PDHX TCL1A ELL GTPBP6 USP9X HIST1H2BO SNHG3 RECK SOAT2 DHX16 GPR65 MAP4K3 CNTNAP1 NDST2 KCNAB2 GAS7 CST7 CAMK1 APOL1 BHLHE40 PDLIM4 SKAP1 ABCB11 S1PR4 TNFRSF25 EED TNFSF14 TNFRSF14 RAB11A TNFRSF6B IL18RAP IL18R1 CD84 KAT2B VNN2 FAM127A FUBP3 P4HA2 F2RL3 MAP3K14 BRSK2 TM4SF5 DOK2 SH2D2A PSTPIP1 SLC7A7 FCGR2C NMI ARHGEF1 FAIM3 IL32 UBE2L6 STK17A CD83 CD163 SLC22A6 LPXN CHST2 AIM2 MAP4K4 ITM2A HOMER1 ARHGEF6 IL27RA PCYT1B THEMIS2 MAGED1 GMFG BRE PREPL CYTIP NFE2L3 ZNF592 IKBKE MICAL2 RALGPS1 FAM53B VGLL4 HERPUD1 KIAA0226 ACAP1 FAM65B RASSF2 SPOCK2 KIAA0125 TESPA1 HEPH ELMO1 TRANK1 NUAK1 LPGAT1 P2RY14 ARHGAP25 GOLGA5 NR1H3 TSPAN32 ARL4A EBI3 CTDSPL KLRG1 CD96 RASGRP2 IGSF6 ZMPSTE24 AKAP8 LILRB2 NMUR1 IKZF1 TRIM22 BTN3A3 BTN2A2 SCML2 ATP8A1 CDIPT IFI30 TACC3 CLEC10A SEMA4D UBD BATF CXCL13 GNLY SORBS1 IFITM2 PDLIM5 TXNIP CELF2 CXCR6 VAMP5 LILRB1 ADAM28 ARID5A FGL2 BTNL3 COPS8 MSL3 IFI44L FERMT2 LILRB4 KDELR3 LILRA2 RAB31 PIM2 ESM1 ADAM30 BTN3A2 BTN3A1 BTN2A1 CD160 CORO1A MAP4K1 SEC63 SP140 TREX1 SLCO2B1 TFEC NTNG1 NLRP1 SEC31A CARD8 KLRK1 PDCD11 MAST3 KIF21B FNBP1 SETX CMTR1 43349 PASK RGL1 RFTN1 CYFIP1 SYT11 FBXO28 NUP210 PLCL2 SEL1L3 CAMSAP2 WWC1 EHBP1 PHF15 SYNE1 SUN1 KIAA0895 ARHGEF18 PPWD1 COTL1 ISCU CBX7 TNFRSF13B ZFYVE26 HMHA1 LILRA4 PRG1 HEBP2 SH3BP4 MAFF APOL2 TNFAIP8 PRKD2 IFFO1 SAMHD1 KBTBD2 MOXD1 IPCEF1 GLCE PPP1R16B CCDC9 FBXW2 PTPN22 STAP1 FBXO3 PLA2G2D GALNT8 CYFIP2 LAT LAMP3 TSPAN13 DKK3 CYTH4 DISC1 GPR162 SIT1 ADAMDEC1 P2RY10 APOBEC3C MAT2B SLCO3A1 IGKV1-5 IGLV2-14 IGLJ3 IGKV1D-13 COA3 TMEM176B CTNNA3 CLEC2D C11orf21 ICOS SNX15 GPR171 FTSJ2 PILRA GLTSCR2 TBX21 CD209 TAX1BP3 PODXL2 IL21R DEF6 CUZD1 TRAT1 C6orf48 CLEC4A NTM DESI2 UBXN1 LAP3 ANGPTL4 INSIG2 LEF1 PLEKHO1 WBP5 HERC5 BET1L TLR7 COA4 GMIP PLAC8 MS4A4A PLA1A ZNF589 TRPV2 NOL7 BIN2 SUCO CHMP3 ACSL5 UIMC1 CD244 TUBA8 CECR1 NUDT9 UBASH3A S1PR5 C21orf91 TREM1 SH3TC1 FAM105A CPVL RHOF EPB41L4B TMEM106B LEPROT ASPN LAX1 TXNL4B HCFC1R1 BANK1 TAPBPL PLEKHJ1 KIAA1551 ZNF532 TMEM140 GIMAP4 SLC29A3 C19orf66 GIMAP5 PI4K2A TMEM176A DNAH3 GALNT10 DOCK10 AMBRA1 BCAS4 IARS2 NECAP2 FAM21B DHX32 WWC3 ARHGAP15 BIN3 MOSPD1 CRTAM SLC2A9 ASCL3 SLAMF8 POGLUT1 PGLYRP4 ATP13A1 RTN4 GRAMD1B MTUS1 BAHCC1 WDR48 SLAMF7 C6orf47 CADM3 CELA2A TNN GPSM3 SAMSN1 RTP4 DPEP2 MS4A6A NARFL CLEC7A ACBD3 PARP12 BCL11B MRPS6 ZNF747 PVRIG PLEKHF1 TMEM243 LILRA6 ZNF576 EFHD2 FCRL2 ZBED2 TNFAIP8L2 PARP8 ANKRD55 ARHGAP28 TREML2 CPSF7 PLBD1 RIN3 DENND1C WLS TMEM156 C14orf159 ATF7IP2 HEXA-AS1 KIAA0226L MUS81 RUNX1-IT1 CXorf21 MED28 TRAF3IP3 THUMPD2 LIMD2 APOL5 APOL3 NPL SLC2A10 FAM49A PLA2G12A ITM2C RASSF4 ZDHHC18 GLYR1 CBR4 GUSBP11 SP140L EGLN3 TOE1 EFHC1 OSBPL9 MBOAT2 MTERFD2 RAB40A A2M-AS1 PYHIN1 DSTNP2 PAOX U2AF1L4 APOBEC3A APOBEC3F GPR116 FGD2 CCZ1B NCR3 SIRPB2 IGKV1OR2-118 GVINP1 LOC391020 MXRA7 CCDC88C IRS3P GIMAP6 NCF1 LOC728392 HLA.A HLA.B HLA.DMA HLA.DMB HLA.DOA HLA.DOB HLA.DPA1 HLA.DPB1 HLA.DQA1 HLA.DQB1 HLA.DRA HLA.DRB1 HLA.E HLA.G KRTAP5.9 IGKV1.5 IGLV2.14 IGKV1D.13 HEXA.AS1 RUNX1.IT1 A2M.AS1 IGKV1OR2.118* |
| ICS5_score | *CXCL13 CLIC5 HLA-F TNFRSF17 XCL2 HLA.F* |
| iDC | *ABCG2 BLVRB CARD9 CD1A CD1B CD1C CD1E CH25H CLEC10A CSF1R CTNS F13A1 FABP4 FZD2 GSTT1 GUCA1A HS3ST2 LMAN2L MMP12 MS4A6A NUDT9 PDXK PPARG PREP RAP1GAP SLC26A6 SLC7A8 SYT17 TACSTD2 TM7SF4 VASH1* |
| IFIT3 | *IFIT3 IFI44L IFIT1 RSAD2 MX1 IFI44 OAS2 CMPK2 IFI6 IFIT2* |
| IFN_21978456 | *IFI27 MX1 ISG15 IFIT1 IFIT3 RSAD2 IFI44L IFI44 OAS1 OAS2 OAS3 HERC6 OASL* |
| IFNG_score_21050467 | *MAP3K10 CSRP3 FOSL1 SKP1A HLA-B SF3A1 XRCC6 CEBPD HLA-A NMI IFI35 HIF3A TAS2R5 C1S SHFM1 COL16A1 PSME1 IFITM1 CYCS BAK1 EPS15 GBP1 FAS PML HADH EIF2B1 HLA-E HADHB IRF1 ADAR IFIT3 SDCBP ATP6V0B ZFP36L2 TRIM21 PRAME ELK4 BBC3 PSMB10 CASP8 PHLDA1 PPP3CA BTG1 RBBP4 PMAIP1 TEAD4 SRP9 ISG15 BST2 PYHIN1 IL15RA VEGFC RHOC PLOD2 NULL STAT1 PSMB8 SFRS2 VAT1 PARP1 ICAM1 TAP1 IL6 BAG1 HLA.B HLA.A HLA.E* |
| IgG_19272155 | *LOC96610 IGKV1OR2-118 IGKV1D-13 IGLL3P IGKV1-5 IGHV1-69 POU2AF1 IGLJ3 GUSBP11 IGLC2 IGLV3-25 IGKC IGLV2-14 IGHG1 IGHM IGLV4-3 IGHD IGHA1 IGL IGH IGLC1 IGKV1OR2.118 IGKV1D.13 IGKV1.5 IGHV1.69 IGLV3.25 IGLV2.14 IGLV4.3* |
| IGG_Cluster_21214954 | *CD27 IL2RG CD79A PIM2 POU2AF1 IGKC IGLV3-25 IGL IGJ TNFRSF17 NTN3 IL8 HLA-C LAX1 IGLV3.25 HLA.C* |
| IL12_score_21050467 | *CD3E TRB IL12B CD3Z STAT4 IFNG JAK2 IL12RB1 IL12RB2 MAPK14 TRA IL12A MAP2K6 TYK2 JUN IL18 CXCR3 CD3G ETV5 CCR5 CD3D MAPK8 IL18R1* |
| IL13_score_21050467 | *IL13 IL13RA2 JAK2 IL13RA1 TYK2 IL4R JAK1* |
| IL2_score_21050467 | *RAF1 IL2 STAT5A IL2RB SOS1 GRB2 STAT5B HRAS LCK JUN MAPK3 IL2RG JAK1 JAK3 CSNK2A1 MAP2K1 FOS SHC1 SYK ELK1 IL2RA MAPK8* |
| IL4_score_21050467 | *JAK3 GRB2 STAT6 SHC1 IL4 IL4R AKT1 IRS1 IL2RG JAK1 RPS6KB1* |
| IL8_21978456 | *CXCL1 CXCL8 CXCL2* |
| Immune_cell_Cluster_21214954 | *WARS IDO1 RNASE6 GPR65 MNDA LAMP3 TNFAIP8 LYN TNFAIP3 PLAC8 IL7R CSF2RB IRF1 TNFRSF1B ADAMDEC1 SLAMF8 SIRPG LTB PTPRCAP CCR7 SPOCK2 PLEK CD74 HLA-DMA HLA-DRA HLA-DMB FCER1G TYROBP LAPTM5 ITGB2 C1QA STAT1 CXCL11 CXCL10 GBP1 TAP1 PSMB9 GIMAP4 SRGN FGL2 HCLS1 ARHGAP25 LCK EVI2B ARHGAP15 CYTIP GZMA LCP2 CD53 IL10RA CD48 CCL5 CD3D CD2 GZMK PTPRC CXCL9 GMFG IRF8 CTSS TFEC SLA SLC7A7 LST1 AIF1 CD86 NCKAP1L THEMIS2 LYZ CD52 SELL HLA-F HLA-B HLA-E GZMB SEMA4D BTN3A2 BTN3A3 CASP1 HLA.DMA HLA.DRA HLA.DMB HLA.F HLA.B HLA.E* |
| Immune_NSCLC_score | *C3orf41 C1orf24 PLEK RHOH PSCDBP TCEA2 FLJ21963 CLEC4E USP51 WFDC10B IGH SLC4A3 CD53 LOC401431 SCFV THRAP2 PRDM13 OBSL1 C7orf40 TAGAP MGC11271 IGLV6-57 CD38 FKBP9 ADAMTSL2 CD48 GNPTAB DHRS8 LOC388886 CNIH3 PSMA6 CCRK SHROOM1 GPSM1 TRO GSTT2 NQO2 EAF2 MUM1L1 MUC4 C13orf21 PABPC1 PLA2G7 PARK2 AOAH IGL LOC642480 TMSB4X LOC390712 ACOT8 GIMAP7 LOC375010 ASAH1 TRIM45 C2orf30 EXT2 IFI6 KCNE3 CTSF SULT1C1 RASL11B LOC148898 HMGCL IGHA1 C1QTNF3 C20orf46 IL11RA ADRA2C IGKC CEACAM5 PURB TPD52 IGLV6.57* |
| Interferon_19272155 | *RSAD2 OAS3 IFI44 ISG15 DDX60 IFI44L IFI6 MX1 IFIT3 IFI27 IFIT1 OAS1 OAS2* |
| Interferon_Cluster_21214954 | *C19orf66 BST2 SP110 PARP12 IFIT5 OAS1 IRF9 USP18 DDX58 MX2 IFI27 OAS2 IFI6 IFIT3 IFI44L IFI44 IFIT1 ISG15 MX1 RSAD2 OAS3 HERC6 HERC5 DDX60 PLSCR1 UBE2L6 SAMD9 RTP4 XAF1 NMI IFITM1 IFITM3 ISG20 PSME2 PSME1 IRF7* |
| IR7_score | *C1QA IGLC2 LY9 TNFRSF17 SPP1 XCL2 HLA-F HLA.F* |
| KEGG_HEMATOPOIETIC_CELL_LINEAGE | *GP1BA GP1BB EPO IL9R CD33 TNF GP9 ITGAM CD34 CD36 GP5 ITGA4 ITGA3 KITLG ITGA2B FCGR1A ITGA5 FCER2 GYPA THPO IL1R2 IL7R MME IL11RA CD19 MS4A1 TFRC CD22 KIT FLT3 ITGB3 FLT3LG IL1A CR2 CD14 CR1 TPO EPOR IL11 CSF2 HLA-DRB4 CD4 CSF1R HLA-DRB5 CSF1 HLA-DRB3 IL3RA IL3 CD59 LOC652799 CD9 CD8A CD8B CD44 CD7 CD5 CSF2RA CD55 DNTT CSF3 IL1B IL1R1 IL2RA ITGA6 IL6R ITGA2 ITGA1 CSF3R IL7 CD37 CD2 CD3D CD3E CD38 CD3G CD1B ANPEP CD1C CD1D CD1E CD1A IL4 IL4R IL5 IL5RA IL6 HLA-DRB1 HLA-DRA HLA.DRB4 HLA.DRB5 HLA.DRB3 HLA.DRB1 HLA.DRA* |
| LCK_19272155 | *ARHGAP15 KLRK1 SASH3 ARHGAP25 SLAMF1 GIMAP5 SH2D1A CCR7 ITK TRBV19 RAC2 CORO1A PIK3CD LPXN PRKCB STAT4 GMFG SELPLG FGL2 EVI2B CSF2RB GZMK SAMSN1 GIMAP4 IRF8 NCKAP1L IL10RA TRAC INPP5D PLAC8 CCL5 CD247 LTB IL2RG HCLS1 IL7R CD53 LCK CD3D CD27 GZMA SELL TRBV21-1 CD2 CD48 SLA LCP2 SRGN PTPRC TRBV21.1* |
| LIexpression_score | *CCL5 CD19 CD37 CD3D CD3E CD3G CD3Z CD79A CD79B CD8A CD8B1 IGHG3 IGJ IGLC1 CD14 LCK LTB MS4A1* |
| LYM | *CD53 SASH3 IL10RA NCKAP1L LCP2 ITGAL CCR5 CD4 MYO1F ARHGAP30* |
| Lymph_vessels | *FIGF PDPN VEGFC* |
| LYMPHS_PCA_16704732 | *UQCRB SESTD1 QTRT1 TIPIN REL STXBP2 HSBP1 COX6C RPL11 MECOM ANKRD28 JUN ZC3H15 RPL23 RPS6KA2 EEF2 TMA7 RPS6 RPL27 RPS21 COX7B PRRC2B CYP17A1 NSUN4 TOMM34 MINOS1 STAMBPL1 FGF9 ATF4 RPL35 RPL31 RPS24 DCLRE1C C5orf49 FAM162A ITGB2 SLC19A1 RPL32 TPP2 MALAT1 LSM3 TSSC1 ATXN2L SERPINB6 TPI1* |
| Macrophages | *APOE ATG7 BCAT1 CCL7 CD163 CD68 CD84 CHI3L1 CHIT1 CLEC5A COL8A2 COLEC12 CTSK CXCL5 CYBB DNASE2B EMP1 FDX1 FN1 GM2A GPC4 KAL1 MARCO ME1 MS4A4A MSR1 PCOLCE2 PTGDS RAI14 SCARB2 SCG5 SGMS1 SULT1C2* |
| Mast_cells | *ABCC4 ADCYAP1 CALB2 CEACAM8 CMA1 CPA3 CTSG ELA2 GATA2 HDC HPGD HPGDS KIT LINC01140 MAOB MLPH MPO MS4A2 NR0B1 PPM1H PRG2 PTGS1 SCG2 SIGLEC6 SLC18A2 SLC24A3 TAL1 TPSAB1 TPSB2 VWA5A* |
| MCD3_CD8_21214954 | *SRPX IGFBP6 ENPP2 SEMA3G CIDEA GPX3 GPD1 CD36 RBP4 AOC3 LPL FABP4 ADIPOQ PLIN1 ADH1B FHL1 LEP CD34 SPRY1 PROS1 PPAP2A AKAP12 JAM3 NDN SPARCL1 ITM2A AQP1 C7 TSPAN7 JAM2 CAV1 GNG11 LDB2 LHFP CDH5 ABCA8 MEOX1 DARC VWF CLDN5 ERG SLIT2 PECAM1 CDO1 CFD* |
| MDACC.FNA.1_20805453 | *TMEM176B SLCO2B1 CKAP2 CD74 VCAM1 IGLL1 CCL2 GPNMB PTGDS CD163 MAFB HLA-DRB1 PLTP APOE C1QB NTN3 PECAM1 IGKV1D-13 IGKV1OR2-108 IGLJ3 IGKC IGL IGHG3 IGHM HLA-DPA1 HLA-DQB1 HLA-DQA1 IGHG1 IGJ C1QA HLA-DRA HLA.DRB1 IGKV1D.13 IGKV1OR2.108 HLA.DPA1 HLA.DQB1 HLA.DQA1 HLA.DRA* |
| MHC.I_19272155 | *HLA-G HLA-A HLA-B HLA-C HLA-F HLA.G HLA.A HLA.B HLA.C HLA.F* |
| MHC.II_19272155 | *CTSS CD74 HLA-DRB1 HLA-DQA1 HLA-DMB HLA-DPA1 HLA-DPB1 HLA-DMA HLA-DRA LCP2 SRGN PTPRC HLA.DRB1 HLA.DQA1 HLA.DMB HLA.DPA1 HLA.DPB1 HLA.DMA HLA.DRA* |
| MHC1_21978456 | *HLA-E HLA-G HLA-B HLA-C HLA-J HLA-F HLA-A HLA.E HLA.G HLA.B HLA.C HLA.J HLA.F HLA.A* |
| MHC2_21978456 | *HLA-DPB1 HLA-DMB CD74 HLA-DRB5 HLA-DRB1 HLA-DRB4 HLA-DRB3 HLA-DRA HLA-DPA1 HLA-DQA1 HLA-DQA2 LOC100509457 HLA-DMA HLA-DQB1 LOC101060835 HLA.DPB1 HLA.DMB HLA.DRB5 HLA.DRB1 HLA.DRB4 HLA.DRB3 HLA.DRA HLA.DPA1 HLA.DQA1 HLA.DQA2 HLA.DMA HLA.DQB1* |
| Minterferon_Cluster_21214954 | *STAT1 IL18BP XAF1 B2M RSAD2 EPSTI1 PSMB8 PSMB9 ZC3HAV1 IFI35 DDX58 GBP7 CXCL10 CCL5 GBP6 CXCL9 CD274 HELZ2 UBA7 LGALS3BP PARP9 IRF9 ADAR IFIT2 OASL DDX60 IFIH1 RTP4 GBP4 IFI44 MX1 ISG15 BST2 USP18 IFIT1 OAS2 ZBP1 PARP14 IRGM STAT2 PARP12 SP100 GBP1 BATF2 NMI SLFN13 GVINP1 IRF1* |
| Module11_Prolif_score | *CDKN3 NDC80 RNASEH2A CENPA SMC2 CENPE RAD51AP1 PLK4 NMU KIF2C TMSB15A UBE2C CHEK1 ZWINT OIP5 CRABP1 ECT2 EIF4EBP1 EZH2 FEN1 HSPA4L TPX2 FOXM1 NCAPH PRAME PDSS1 KIF4A RAD54B ASPM FBXO5 ATAD2 RACGAP1 GPSM2 DONSON HMMR BIRC5 KIF11 LMNB1 MAD2L1 MCM4 MCM5 MKI67 MMP1 MYBL1 MYBL2 NEK2 NUSAP1 GTSE1 GINS2 PLK1 FAM64A ERCC6L NCAPG2 CEP55 FANCI HJURP MCM10 DEPDC1 C1orf112 CENPN PBK KIF15 CIAPIN1 ACTR3B GPR126 SPC25 RAD21 RFC3 RFC4 RRM2 NCAPG STIL SKP2 SOX11 SQLE AURKA TAF2 TARS BUB1B TK1 TMPO TOP2A PHLDA2 TTK LRP8 DSCC1 MLF1IP E2F8 SHCBP1 SLC7A5 ANP32E KIF18A CDC7 CDC45 RAD54L TTF2 PIR ACTL6A GGH CCNA2 CCNB1 PRC1 CCNB2 CCNE2 EXO1 AURKB PTTG1 TRIP13 KIF23 APOBEC3B MTFR1 ESPL1 DLGAP5 CDK1 MELK GINS1 CDC6 CDC20 NCAPD2 KIF14* |
| Module3_IFN_score | *IFI44 IFI44L DDX58 IFI6 IFI27 IFIT2 IFIT1 IFIT3 CXCL10 MX1 OAS1 OAS2 OAS3 HERC5 SAMD9 HERC6 DDX60 RTP4 IFIH1 STAT1 TAP1 OASL RSAD2 ISG15* |
| Module4_TcellBcell_score | *CD96 CD52 SEMA4D CXCL13 SP140 CCR7 CTSW DOCK2 EVI2B FCN1 KLRK1 FLI1 PLCL2 FYB IPCEF1 PPP1R16B CCDC69 STAP1 GPR18 ICOS GPR171 GZMA GZMB GZMK IGJ IL2RB IL2RG IL7R ITGA4 ITK KLRB1 LCK LGALS2 LRMP LTB SH2D1A CXCL9 NCF4 GIMAP6 IL21R TRAT1 PLAC8 UBASH3A POU2AF1 RHOF LAX1 BANK1 SIRPG PRF1 DOCK10 PRKCB CRTAM PTGDS PTPRC PTPRCAP TNFRSF17 CCL19 SELL BCL11B SLAMF1 TNFRSF1B CCR2 TRAF3IP3 TCL1A VNN2 PSTPIP1 CD2 CD3G CD247 CD7 CD8A CD19 MS4A1 CD27 AIM2 CD37 CYTIP CD69 CD79A FAM65B KIAA0125 P2RY14* |
| Module5_TcellBcell_score | *IGSF6 LILRB2 BTN3A3 UBD CXCL13 GNLY CXCR6 CTSC HCP5 PIM2 SP140 CCR7 CTSS CYBB FCN1 TFEC SEL1L3 FYB GBP1 LAMP3 ADAMDEC1 GPR18 ICOS GPR171 GZMH GZMB GZMK BIRC3 IFNG IL2RG IL15 IDO1 CXCL10 IRF1 ISG20 ITK LAG3 LCK LYN CXCL9 NKG7 TRAT1 MGC29506 PLAC8 POU2AF1 CRTAM SLAMF8 PSMB9 PTPN7 SLAMF7 BCL2A1 TNFRSF17 CCL5 CCL8 CCL13 CCL18 CCL19 CXCL11 SELL SAMSN1 RTP4 CLEC7A TAP1 WARS PLA2G7 ZBED2 NPL RUNX3 VNN2 CD3G IL32 CD8B CD19 CD86 AIM2 CD38 CYTIP LOC96610 CD69 CD79A* |
| Neutrophils | *ALPL BST1 CD93 CEACAM3 CREB5 CRISPLD2 CSF3R CYP4F3 DYSF FCAR FCGR3B FLJ11151 FPR1 FPRL1 G0S2 HIST1H2BC HPSE IL8RA IL8RB KCNJ15 LILRB2 MGAM MME PDE4B S100A12 SIGLEC5 SLC22A4 SLC25A37 TECPR2 TNFRSF10C VNN3* |
| NHI_5gene_score | *IGK GBP1 STAT1 IGLL5 OCLN* |
| NK_CD56bright_cells | *DUSP4 FOXJ1 LPCAT4 MADD 43165 MPPED1 MUC3B NIBP PLA2G6 RRAD XCL1* |
| NK_CD56dim_cells | *EDG8 FLJ20699 GTF3C1 GZMB IL21R KIR2DL3 KIR2DS1 KIR2DS2 KIR2DS5 KIR3DL1 KIR3DL2 KIR3DL3 KIR3DS1 SPON2 TMEPAI* |
| NK_cells | *ADARB1 AF107846 ALDH1B1 APBB2 ATL2 BCL2 CDC5L FGF18 FUT5 FZR1 GAGE2A IGFBP5 KANK2 LDB3 MAPRE3 MCM3AP MRC2 NCR1 PDLIM4 PRX PSMD4 RP5-886K2.1 SGMS1 SLC30A5 SMEK1 SPN TBXA2R TCTN2 TINAGL1 TRPV6 XCL1 XCL2 ZNF205 ZNF528 ZNF747 RP5.886K2.1* |
| PD1_data | *PDCD1* |
| PD1_PDL1_score | *PDCD1 CD274* |
| pDC | *IL3RA* |
| PDL1 | *CD274* |
| PDL1_data | *CD274* |
| Rotterdam_ERneg_PCA_15721472 | *PARP4 COL2A1 FUT3 GAS2 MYH2 SAT1 TNFSF10 CEP57 ANAPC15 GABRQ RFX7 BCL2L14 RPL23AP7 ZNF362* |
| STAT1_19272155 | *TAP1 GBP1 IFIH1 PSMB9 CXCL9 IRF1 CXCL11 CXCL10 IDO1 STAT1* |
| STAT1_score | *STAT1 CXCL10 TAP1 CXCL11 INDO CXCL9 MX1 LAMP3 ISG15 RTP4 HERC6 IFI44L MX2 IFIT3 HERC5 RSAD2 DDX58 CCL5 ADAMDEC1 CD2 HCP5 NMI SPOCK2 CCL8 TRIM22 LYZ IRF1 LAG3 PSCDBP TFEC UBD SP140 CTSC IFI6 PLA2G7 CD3G ECGF1 PLAC8 FGL2 GZMK CD48 STAT4 GPR18 P2RX5 IFI30 SH2D1A LAPTM5 CD69 PTPN7 IRF8 PIM2 ETV7 GPR171 PSME1 BIRC3 FASLG IFITM1 IFIT5 ITGB2 BTN3A2 HCLS1 SECTM1 ARHGAP15 KLRK1 IGSF6 EBI2 SNX10 BST2 APOC1 ZC3HAV1 DDAH2 LILRA4 EBI3 KLRC3 CLEC4A CD40LG VAV1 GLRX ACP5 RFX5 CECR1 TRAF3 RAB8A IL18 EFNA1 RASGRP1 REC8L1 CCRL2 DNAL4* |
| T_cell_PCA_16704732 | *SLC35D2 CD3E S100A8 GATA3 GIPC1 CCL5 PRKCA SLCO3A1 CEP85L KLRB1 CD5 SH2D1A S100A10 IL6ST CD247 CD6 SBK1 GBP2 KLRG1 TIAM1 MYBL1 TACC3 RASGRP1 LAT OSBP2 LIMA1 UPP1 RNF213 MAPKAPK5 ACVR2B FYB HSPA1L LEF1 MRPL27 LRIG1 RARRES3 DPP4 TRA APBA2 SPOCK2 MLLT3 PCSK5 BCL11B VIPR1 RUNX2 GALT ITGA6 OLAH CD3G DNASE1L3 CAMK4 DNAJB1 KLRC4 TNFSF8 NSG1 SATB1 GZMK IL18R1 IFITM1 LYAR CISH PRSS1 PRKCI BIN2 TRAT1 SORL1 CD3D PIK3IP1 PCYT2 CCND2 CTSW LINS ITK DISC1 STAT4 MAN1C1 ITM2A MAP7D1 TCF7 TOMM40 PKM SYT1 NAP1L5 SHFM1 LPAR6 MATN2 NR4A2 SLFN5 TOB1 CD28 PDE9A TMEM173 DOCK9 MORC2-AS1 AOC1 PTGER2 LCP2 RORA AKTIP NELL2 SPEG LPIN2 RTKN2 FHIT PDE4D SLC39A8 SELPLG SYNE2 LEPROTL1 BUB1B ACTN1 AQP3 PRKCQ RSU1 GPSM3 TSEN54 SOCS3 GABARAPL1 C15orf62 FYN ATP1A1 NGFRAP1 DUSP16 GBP1 RGS10 INPP4B TRABD2A RBMS1 MAL RAB43 ATP13A4 FAM134B C20orf112 ITPKB DUSP2 TNIK LPAR2 IL32 APBB1 ARL4C ID2 PRKCQ-AS1 TNFRSF25 HOXB2 CD2 ADA JAKMIP1 MGAT4A FAM102A RMDN1 WWP1 TARP NPTXR TESPA1 LDHB PXN FBLN5 TNFAIP3 WNT10B LCK PIK3R1 MEN1 FLT3LG NPDC1 ANXA1 TXK MPP7 IL7R ZAP70 CEP41 CAPZB CDC14A SNPH MORC2.AS1 PRKCQ.AS1* |
| T_cells | *BCL11B CD2 CD28 CD3D CD3E CD3G CD6 CD96 GIMAP5 ITM2A LCK NCALD PRKCQ SH2D1A SKAP1 TRA TRAC TRAT1 TRBC1* |
| T_helper_cells | *ANP32B ASF1A ATF2 BATF C13orf34 CD28 DDX50 FAM111A FRYL GOLGA8A ICOS ITM2A LRBA NAP1L4 NUP107 PHF10 PPP2R5C RPA1 SEC24C SLC25A12 SRSF10 TRA UBE2L3 YME1L1* |
| TAMsurr_score | *CXCL10 CXCL11 CCL8 LAMP3* |
| TAMsurr_TcClassII_ratio | *CXCL10 CXCL11 CCL8 LAMP3 CD2 CD3G CD8A IFNG TNF GZMB GZMH PRF1 ZAP70 HLA-DMA HLA-DOA HLA-DOB HLA-DPA1 HLA-DPB1 HLA-DQA1 HLA-DQB1 HLA-DQB2 HLA-DRA HLA-DRB1 HLA-DRB2 HLA-DRB3 HLA-DRB4 HLA-DRB5 HLA-DRB6 CIITA CD74 HLA.DMA HLA.DOA HLA.DOB HLA.DPA1 HLA.DPB1 HLA.DQA1 HLA.DQB1 HLA.DQB2 HLA.DRA HLA.DRB1 HLA.DRB2 HLA.DRB3 HLA.DRB4 HLA.DRB5 HLA.DRB6* |
| TcClassII_score | *CD2 CD3G CD8A IFNG TNF GZMB GZMH PRF1 ZAP70 HLA-DMA HLA-DOA HLA-DOB HLA-DPA1 HLA-DPB1 HLA-DQA1 HLA-DQB1 HLA-DQB2 HLA-DRA HLA-DRB1 HLA-DRB2 HLA-DRB3 HLA-DRB4 HLA-DRB5 HLA-DRB6 CIITA CD74 HLA.DMA HLA.DOA HLA.DOB HLA.DPA1 HLA.DPB1 HLA.DQA1 HLA.DQB1 HLA.DQB2 HLA.DRA HLA.DRB1 HLA.DRB2 HLA.DRB3 HLA.DRB4 HLA.DRB5 HLA.DRB6* |
| Tcell_21978456 | *CORO1A LCK GZMK LAPTM5 IL10RA CD27 SELL TRAC CD48 CD2 TRBC1 CD3D IL23A ITK CD53 EVI2B PTPRC YME1L1 TRAV20 TRAJ17 SAMSN1 ARHGAP25 PLAC8 CD52* |
| Tcell_receptors_score | *CD3D CD3E CD3G CD3Z* |
| Tcm_cells | *AQP3 ATF7IP ATM CASP8 CDC14A CEP68 CLUAP1 CREBZF CYLD DOCK9 FAM153B FOXP1 FYB HNRPH1 INPP4B KLF12 LOC441155 MAP3K1 MLL N4BP2L2-IT2 NEFL NFATC3 PCM1 PCNX PDXDC2 PHC3 POLR2J2 PSPC1 REPS1 RPP38 SLC7A6 SNRPN ST3GAL1 STX16 TIMM8A TRAF3IP3 TXK TXLNGY USP9Y N4BP2L2.IT2* |
| Tem_cells | *AKT3 C7orf54 CCR2 DDX17 EWSR1 FLI1 GDPD5 LTK MEFV NFATC4 PRKY TBC1D5 TBCD TRA VIL2* |
| Tfh_cells | *B3GAT1 BLR1 C18orf1 CDK5R1 CHGB CHI3L2 CXCL13 HEY1 HIST1H4K ICA1 KCNK5 KIAA1324 MAF MAGEH1 MKL2 MYO6 MYO7A PASK PDCD1 POMT1 PTPN13 PVALB SH3TC1 SIRPG SLC7A10 SMAD1 ST8SIA1 STK39 THADA TOX TSHR ZNF764* |
| Tgd_cells | *C1orf61 CD160 FEZ1 TARP TRD TRGV9* |
| TGFB_PCA_17349583 | *COL1A1 CTGF FN1 MMP9 SERPINE1 SPARC TAGLN TGFB1 TGFB1I1 TGFB3 TGFBI TGFBR1 TGIF1 THBS1 TGIF2* |
| TGFB_score_21050467 | *MMP3 MARCKSL1 IGF2R LAMB1 SPARC FN1 ITGA4 SMO MMP19 ITGB8 ITGA5 NID1 TIMP1 SEMA3F RHOQ CTNNB1 MMP2 SERPINE1 EPHB2 COL16A1 EPHA2 TNC JUP ITGA3 TCF7L2 COL3A1 CDH6 WNT2B ADAM9 DSP HSPG2 ARHGAP1 ITGB5 IGFBP5 ARHGDIA LRP1 IGFBP2 CTNNA1 LRRC17 MMP14 NEO1 EFNA5 ITGB3 EPHB3 CD44 IGFBP4 TNFRSF1A RAC1 PXN PLAT COL8A1 WNT8B IGFBP3 RHOA EPHB4 MMP1 PAK1 MTA1 THBS2 CSPG2 MMP17 CD59 DVL3 RHOB COL6A3 NOTCH2 BSG MMP11 COL1A2 ZYX RND3 THBS1 RHOG ICAM1 LAMA4 DVL1 PAK2 ITGB2 COL6A1 FGD1* |
| Th1_cells | *APBB2 APOD ATP9A BST2 BTG3 CCL4 CD38 CD70 CMAH CSF2 CTLA4 DGKI DOK5 DPP4 DUSP5 EGFL6 GGT1 HBEGF IFNG IL12RB2 IL22 LRP8 LRRN3 LTA SGCB SYNGR3 ZBTB32* |
| Th17_cells | *IL17A IL17RA RORC* |
| Th2_cells | *ADCY1 AHI1 ANK1 BIRC5 CDC25C CDC7 CENPF CXCR6 DHFR EVI5 GATA3 GSTA4 HELLS IL26 LAIR2 LIMA1 MB MICAL2 NEIL3 PHEX PMCH PTGIS SLC39A14 SMAD2 SNRPD1 WDHD1* |
| Treg_cells | *FOXP3* |
| TREM1_data | *TREM1* |
| Troester_WoundSig_19887484 | *ADH1A APOH BMPR1B DST CAPN6 CD86 CDK4 COL17A1 COX7B CSF3 CTGF CTSK DMBT1 ARID3A DSC3 ELANE EYA4 F3 FABP4 FAP FBLN1 EFEMP1 FPR1 FRZB GJB5 HBB HDC HMGA1 HPCAL1 CYR61 IL1B IL13RA2 IDO1 INHBA KRT6A KRT6B KRT34 AFF3 LAMA3 LAMC2 LTBP2 LUM MSMB ND4 NODAL OGN PCDH8 PDGFRL PER1 PFKFB3 PITX1 PML PNLIPRP2 S100A8 SAA4 CCL2 TGFB2 THBS1 TLR2 UGT2B17 ZIC2 TFPI2 NR4A3 SPARCL1 TP63 PER2 SPAG9 CXCL14 TMCC2 TNFSF15 CLEC4M NPM2 HOXB13 PPP6R1 SULF1 DICER1 ABI3BP EGFL6 TIPARP RGS22 IGLV2-14 A1CF TREM1 PGPEP1 RGMA ADAMTS9 TENM2 MKL2 VPS18 CACHD1 GUF1 ELSPBP1 ZBED2 LRRC2 CHPF HMBOX1 TMC5 BAALC APOLD1 VMP1 SPACA1 FRMD8P1 FAM71F1 TNS4 KIAA1751 CROCCP3 OSR2 LRR1 TMEM139 CCBE1 FAM19A4 BMPER YPEL4 JAKMIP3 LOC284454 KRT6C ZNF713 SIMC1 LOC389332 SPDYE8P LOC440934 MUC5B C13orf45 KRT17P3 LOC100131138 IGLV2.14* |
| ZHANG_INTERFERON_RESPONSE | *IRF1 IFI27 IFRD1 STAT1 MX2 OAS1 OASL ISG15 MX1 IFIT2 SP110 IFITM3 OAS2 IFNB1 IRF9 IFI44 IFITM1 ISG20 IFI16 OAS3 IFI30 IRF3 RTP4* |
