# Supplementary material for: An immunomodulatory signature of responsiveness to immune checkpoint blockade therapy
Source: Clin Transl Med. 2020 Dec 21;10(8):e238. doi: 10.1002/ctm2.238 (PMC7752154; doi:10.1002/ctm2.238)
Supplement: Supplementary file 2 — Table S1. Data source information. Table S2. Immunomodulatory gene sets. Table S3. Clinical characteristic of the patients with ICB therapy in two clinical trials. Table S4. Immune related signaling gene sets. [file CTM2-10-e238-s002.docx]

**Method**

**Data collection and processing**

We obtained gene expression data of single-cells from 10 previous studies (**Table S1**) that encompassed melanoma, head and neck carcinoma, colorectal, non-small cell lung cancer, liver cancer, gliomas and astrocytoma, all of which were profiled by Smart-Seq2. In addition, we curated pre-treatment gene expression and clinical data from two ICB therapy clinical trials each with more than 100 samples^1,2^. We normalized the gene expression matrix by log2 transformation and scaled each gene by subtracting its mean and dividing with standard deviation.

**Dissecting single cell expression signature**

We used contrastive learning to learn gene expression signatures of single cells. In this study, contrastive learning was used to learn similar representation of single cell expression by narrowing the gap between the augmented and corresponding original gene expression profiles. The deep learning model that we developed is based on Momentum Contrast algorithm^3,4^. This deep learning framework consisted of a deep neural network as feature encoder and multi-layer perceptron (MLP) as project head to map features learned by the encoder network to space where contrastive learning is applied. We used a deep neural network of 63 layers with dense connection^5^ as feature encoder. This feature encoder has 258 output units. The use of MLP as project head has demonstrated to be beneficial for unsupervised feature learning^6^. We used data augmentation to increase data diversity and mimic data variation. The data augmentation operations include random shuffling or zeroing out 20% of gene expression values. We used stochastic gradient descent algorithm to train the model in parallel on two graphic processing units for 300 epochs with an initial learning rate of 0.24, weight decay of 0.0001 and batch size of 256. The learning rate was decayed by 0.1 at epoch 150 and 250. We developed the model with PyTorch package (v1.3.0).

**Extraction of gene expression signature**

We extracted gene expression signatures for single cells via the aforementioned self-supervised deep learning model. We applied fast interpolation-based t-SNE (t-Distributed Stochastic Neighbor Embedding) to transform these signatures into two-dimensional embedding^7^. We dichotomized each expression signature with cutoff of zero into two clusters: a score > 0 was considered to be activated. Together with the normalized expression matrix, this binary label was subsequently used as input to R routine FindMarkers implemented in Seurat package (v3.1.5) to identify significantly differentially expressed marker genes. A differential expressed marker gene was required to satisfy these criteria: absolute value of log-transformed fold-change > 0.1 and adjusted p-value < 0.01. We used log-transformed fold-change as statistic to perform gene set enrichment analysis (GSEA) for a gene set involved in immunomodulation, which was further categorized into co-simulator, co-inhibitor, ligand, receptor, cell adhesion, antigen presentation and others^8^, respectively. Signatures were ranked according to enrichment score of GSEA. We examined the association of top-5 ranking expression signatures with ICB therapy response.

**Association of top-5 ranking expression signatures with ICB therapy response**

We grouped ICB therapy response into clinical improvement and no clinical improvement. The former was defined as Complete Response (CR) or Partial Response (PR), whereas the latter as Stable Disease (SD) or Progressive Disease (PD). We extracted the corresponding top-5 expression signatures obtained from single cell data for samples in the two ICB therapy clinical trials. These five expression signatures were also dichotomized by a cutoff of zero. We assessed the association of these signatures with ICB therapy response using Fisher’s exact test. Subsequently, we examined the significant signatures obtained from Fisher’s exact test in multivariate logistic model by controlling for tumor mutation burden, the degree of cytotoxic lymphocytes infiltration, levels of *PD-1*, *PD-L1* and *CTLA-4*. We performed multivariate logistic model with stan_lm routine that was implemented in R package Rstanarm (v 2.19.3). We used the average gene expression level of *CD8A*, *CD8B*, *GZMA*, *GZMB* and *PRF1* as surrogate of cytotoxic lymphocytes infiltration. We considered a tumor to be TMB-high if the total number of exomic mutations > 16 mutations per mega base^9^. We dichotomized the expression levels of *PD-1*, *PD-L1* and *CTLA-4* by their corresponding median value in the multivariate logistic model.

**Statistical analysis**

We used CIBERSORT algorithm to decipher tumor immune microenvironment using the LM22 signature^10^. GSEA was conducted with R package fgsea (v 1.6.0). Kaplan-Meier survival and multivariate Cox regression analyses were used to analyze the relationship between gene expression signatures and prognosis by R package survival (v 3.1.12). We used the log-rank test to calculate differences of survival curves. Benjamini-Hochberg procedure was used to adjust p-values for multiple hypothesis test when appropriate. Two-sided test was used if not specified.

**References:**

1. Mariathasan S, Turley SJ, Nickles D, et al. TGFbeta attenuates tumour response to PD-L1 blockade by contributing to exclusion of T cells. Nature 2018;554:544-8.

2. Liu D, Schilling B, Liu D, et al. Integrative molecular and clinical modeling of clinical outcomes to PD1 blockade in patients with metastatic melanoma. Nat Med 2019;25:1916-27.

3. Momentum Contrast for Unsupervised Visual Representation Learning. 2019.

4. Improved Baselines with Momentum Contrastive Learning. 2020.

5. Densely Connected Convolutional Networks. 2016.

6. A Simple Framework for Contrastive Learning of Visual Representations. 2020.

7. Linderman GC, Rachh M, Hoskins JG, Steinerberger S, Kluger Y. Fast interpolation-based t-SNE for improved visualization of single-cell RNA-seq data. Nat Methods 2019;16:243-5.

8. Thorsson V, Gibbs DL, Brown SD, et al. The Immune Landscape of Cancer. Immunity 2018;48:812-30 e14.

9. Gandara DR, Paul SM, Kowanetz M, et al. Blood-based tumor mutational burden as a predictor of clinical benefit in non-small-cell lung cancer patients treated with atezolizumab. Nat Med 2018;24:1441-8.

10. Newman AM, Liu CL, Green MR, et al. Robust enumeration of cell subsets from tissue expression profiles. Nat Methods 2015;12:453-7.
